# Brain age predicts long-term recovery in post-stroke aphasia

**DOI:** 10.1093/braincomms/fcac252

**Published:** 2022-10-06

**Authors:** Sigfus Kristinsson, Natalie Busby, Christopher Rorden, Roger Newman-Norlund, Dirk B den Ouden, Sigridur Magnusdottir, Haukur Hjaltason, Helga Thors, Argye E Hillis, Olafur Kjartansson, Leonardo Bonilha, Julius Fridriksson

**Affiliations:** Center for the Study of Aphasia Recovery, University of South Carolina, Columbia, SC 29208, USA; Center for the Study of Aphasia Recovery, University of South Carolina, Columbia, SC 29208, USA; Center for the Study of Aphasia Recovery, University of South Carolina, Columbia, SC 29208, USA; Department of Psychology, University of South Carolina, Columbia, SC 29208, USA; Center for the Study of Aphasia Recovery, University of South Carolina, Columbia, SC 29208, USA; Department of Psychology, University of South Carolina, Columbia, SC 29208, USA; Center for the Study of Aphasia Recovery, University of South Carolina, Columbia, SC 29208, USA; Department of Communication Sciences and Disorders, Columbia, SC 29208, USA; Department of Medicine, University of Iceland, Reykjavik 00107, Iceland; Department of Medicine, University of Iceland, Reykjavik 00107, Iceland; Department of Neurology, Landspitali University Hospital, Reykjavik 00101, Iceland; Department of Medicine, University of Iceland, Reykjavik 00107, Iceland; Center for the Study of Aphasia Recovery, University of South Carolina, Columbia, SC 29208, USA; Department of Neurology, Johns Hopkins University School of Medicine, Baltimore, MA 21218, USA; Department of Neurology, Landspitali University Hospital, Reykjavik 00101, Iceland; Center for the Study of Aphasia Recovery, University of South Carolina, Columbia, SC 29208, USA; Department of Neurology, Medical University of South Carolina, Charleston, SC 29425, USA; Center for the Study of Aphasia Recovery, University of South Carolina, Columbia, SC 29208, USA; Department of Communication Sciences and Disorders, Columbia, SC 29208, USA

**Keywords:** aphasia, neuroimaging, ageing, age, stroke

## Abstract

The association between age and language recovery in stroke remains unclear. Here, we used neuroimaging data to estimate brain age, a measure of structural integrity, and examined the extent to which brain age at stroke onset is associated with (i) cross-sectional language performance, and (ii) longitudinal recovery of language function, beyond chronological age alone. A total of 49 participants (age: 65.2 ± 12.2 years, 25 female) underwent routine clinical neuroimaging (T1) and a bedside evaluation of language performance (Bedside Evaluation Screening Test-2) at onset of left hemisphere stroke. Brain age was estimated from enantiomorphically reconstructed brain scans using a machine learning algorithm trained on a large sample of healthy adults. A subsample of 30 participants returned for follow-up language assessments at least 2 years after stroke onset. To account for variability in age at stroke, we calculated proportional brain age difference, i.e. the proportional difference between brain age and chronological age. Multiple regression models were constructed to test the effects of proportional brain age difference on language outcomes. Lesion volume and chronological age were included as covariates in all models. Accelerated brain age compared with age was associated with worse overall aphasia severity (F(1, 48) = 5.65, *P* = 0.022), naming (F(1, 48) = 5.13, *P* = 0.028), and speech repetition (F(1, 48) = 8.49, *P* = 0.006) at stroke onset. Follow-up assessments were carried out ≥2 years after onset; decelerated brain age relative to age was significantly associated with reduced overall aphasia severity (F(1, 26) = 5.45, *P* = 0.028) and marginally failed to reach statistical significance for auditory comprehension (F(1, 26) = 2.87, *P* = 0.103). Proportional brain age difference was not found to be associated with changes in naming (F(1, 26) = 0.23, *P* = 0.880) and speech repetition (F(1, 26) = 0.00, *P* = 0.978). Chronological age was only associated with naming performance at stroke onset (F(1, 48) = 4.18, *P* = 0.047). These results indicate that brain age as estimated based on routine clinical brain scans may be a strong biomarker for language function and recovery after stroke.

## Teaser

Kristinsson *et al*. examined the association between estimated brain age, a measure of cortical atrophy derived from clinical-grade brain scans, and language recovery after acute left hemisphere stroke. Primary findings revealed a strong association between brain age and language function at stroke onset as well as long-term recovery of language.

## Graphical Abstract

**Graphical abstract:**
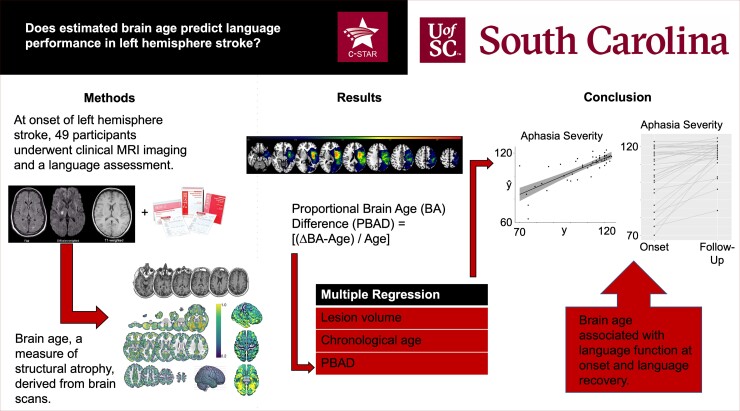

## Introduction

Aphasia is a language impairment that is generally recognized as one of the most disabling consequences of a stroke affecting the language-dominant brain hemisphere.^1^ Most individuals with aphasia recover some language functions in the days and months following the stroke,^2^ but the factors associated with recovery remain poorly understood.^3^ Prior studies indicate that the initial severity of aphasia,^4,5^ the size of the cortical infarct,^6,7^ and the lesion site^6,8^ account for substantial variability in long-term outcomes.

The relationship between recovery and other variables such as age is less clear.^9^ Neuroplastic properties of the brain decrease with age,^10,11^ suggesting that age might be an important factor in aphasia recovery. However, older individuals are more likely to present with severe aphasia,^1,2^ which may indicate that the effect of age on recovery is confounded by aphasia severity, at least in some age groups. Recent research suggests that brain age, which is based on an estimate of cortical tissue integrity, is a more useful indicator of neuroplastic properties of the brain.^12,13^ In the current study, we report the first acute-to-chronic examination of the impact of estimated brain age for longitudinal language recovery in aphasia.

Healthy ageing is accompanied by reliable changes to structural integrity of the brain; in particular, atrophy of grey matter, reduced volume of white matter connections, and distorted functional connectivity have been observed with magnetic resonance imaging.^14–22^ The recently coined concept of *brain age* broadly represents these changes.

Brain age is generally predicted using machine learning algorithms that leverage neuroimaging-derived measures of structural atrophy to estimate how old the brain looks compared to a large sample of healthy control subjects.^12^ The extent to which brain age deviates from chronological age has been found to be associated with onset of psychiatric and neurologic diseases,^13,23^ physical functioning,^24,25^ and cognitive abilities.^24,26–28^ This suggests that estimated brain age may potentially be implemented as a surrogate measure for cognitive reserve.

The presence of a brain lesion presents a challenge for the estimation of brain age since current approaches depend on the quality of normalization of the neuroimages, i.e. warping individual brains into standard space. Necrotic brain tissue can markedly distort the normalization, which is generally designed to process healthy brain images.^29^ This issue can be bypassed by applying an enantiomorphic algorithm to native T_1_ images to effectively ‘heal’ the damaged hemisphere.^30^ The enantiomorphic ‘healing’ takes advantage of the left-right symmetry across hemispheres to replace tissue in the damaged hemisphere with a mirror image of healthy tissue from the contralateral hemisphere. This approach has been successfully applied in our prior work,^31–34^ as well as by other groups.^35–37^

The rate of brain atrophy is increasingly implemented as a clinical biomarker in various neurological disorders characterized by a marked deviation between brain age and chronological age.^38–40^ In the context of stroke, recent studies have emphasized the detrimental impact of stroke as manifested in accelerated brain age.^41,42^ Others have observed an association between brain age and stroke risk,^43^ potentially indicating that biological brain age may both be a biomarker for stroke risk and exacerbated as a consequence of brain damage. Critically, while the association between neurodegeneration and cognitive function has been observed in many neurological disorders,^23,26,44^ the relationship between brain age and cognitive outcomes in post-stroke functional recovery remains to be studied in detail.^45–48^

To this end, we examined the association between brain age and language outcomes after stroke. Specifically, we tested the hypothesis that brain age at stroke onset is associated with: (i) cross-sectional language function and (ii) long-term recovery of language function, *beyond chronological age*. We expected accelerated brain age relative to chronological age to be associated with poorer language function and worse recovery. This study leveraged retrospective clinical neuroimaging data and language assessments collected at stroke onset and at follow-up least 2 years after stroke onset.

## Methods

### Participants

A total of 49 individuals with acute left hemisphere injury were included in the study. Participants were recruited through the neurology ward at the National University Hospital of Iceland, Reykjavk. Participants were eligible for inclusion in the study if they (i) had incurred a single, unilateral left hemisphere stroke, (ii) were in the acute phase of recovery, (iii) had their stroke confirmed by a CT/MRI scan, (iv) had no history of prior stroke, major psychiatric illness or other neurologic impairment affecting the brain, (v) were native speakers of Icelandic, and (vi) gave informed consent for study participation. All study procedures were approved by the Institutional Review Board of the University of Iceland. For a detailed description of participants and procedures, see Magnusdottir *et al.*^49^ and Kristinsson *et al.*^50^

**Figure 1:**
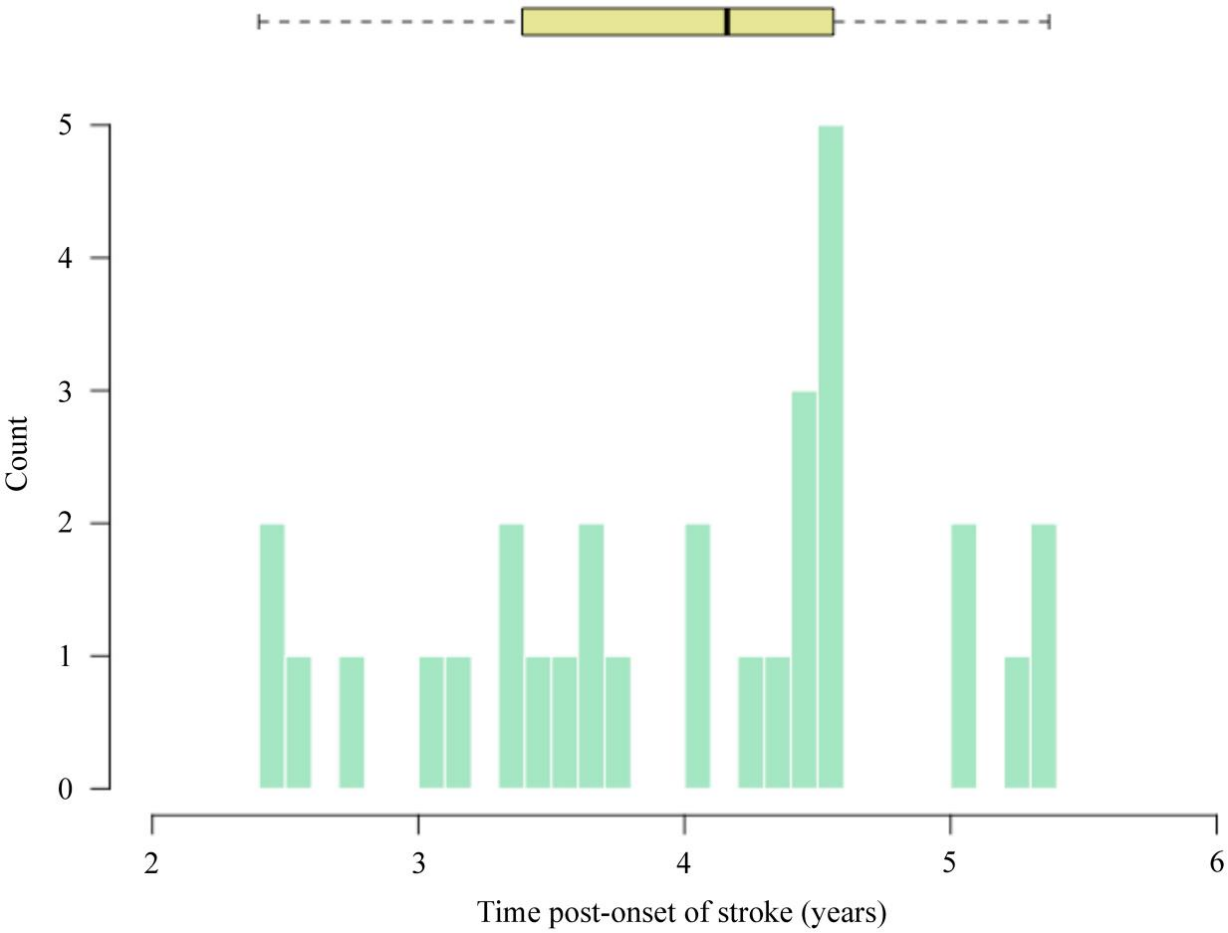
**Time post-onset.** TPO of left hemisphere stroke in years at retesting (min = 2.4y, max = 5.4y; *N* = 30). The boxplot shown at the top of the figure represents median TPO, quartiles and whiskers denote the range of values.

Participants underwent MRI and language assessments within three days of hospital admission. At stroke onset, the average age of the sample was 65.2 years (SD = 12.2 years, range: 34–85 years) and 25 participants were female. A subsample of 30 participants returned for a follow-up language assessment at least 24 months post-onset. At the time of retesting, the average age of the sample was 67.5 years (SD = 10.2 years, range: 43–82 years). The range of time post-stroke across participants was 2.4–5.4 years (mean = 4.0 years, SD = 0.9 years) at retesting. Figure 1 shows time post-onset (TPO) across participants.

### Language assessments

Speech and language impairment was assessed with the Bedside Evaluation Screening Test-Second Edition (BEST-2).^51^ The BEST-2 is designed to assess language function at bedside in acute patients who may not be able to complete a full language assessment battery. In addition to providing an assessment of overall language impairment (henceforth, overall score), the BEST-2 assesses several language domains, including naming, speech repetition, and auditory comprehension. As these domains correspond to the main subtests on the Western Aphasia Battery-Revised,^52^ which is the most widely used aphasia test,^53^ we included all four scores in the data analyses. Importantly, despite being a short evaluation, our prior work has shown that the BEST-2 is sensitive to language impairment and longitudinal changes in language function.^49,50,54^

### MRI

MRI data were collected as part of routine clinical care in acute stroke on a 1.5 T Siemens scanner. We obtained T_1_-weighted images, diffusion-weighted imaging (DWI), and fluid-attenuated inversion recovery (FLAIR) scans. The details for these sequences are as follows: *T_1_-weighted image* [3D GR\IR sequence, repetition time (TR) = 1160 ms, inversion time (TI) = 600 ms, echo time (TE) = 4.24 ms, flip angle = 15°, the 256 × 256 matrix was reconstructed at 512 × 512, yielding a 0.45 × 0.45 mm^2^ in axial-plane resolution, with 192 0.9 mm slices), *diffusion-weighted images* (three scans with B0 = 0, 500, and 1000; TR = 3808 ms, TE = 89 ms, flip angle = 90^o^, Nx = 4, 192 × 192 matrix, 1.2 × 1.2 mm^2^ in axial plane, 24 slices, each 5 mm thick with 1.5 mm gap), and *T_2_-weighted FLAIR image* (TR = 9000 ms, TI = 2500 ms, TE = 112 ms, flip angle = 15°, 280 × 320 matrix with 0.72 × 0.72 mm^2^ in axial-plane resolution, 24 slices, each 5 mm thick with 1.5 mm gap). Images were converted from DICOM to NIfTI format using dcm2niix,^55^ which preserves spatial coordinates (yielding a good starting estimate for the subsequent co-registration of the T_1_ image to the T_2_ scan).

An expert neurologist or trained study staff member with extensive experience/training in lesion drawing manually demarcated the brain lesions on FLAIR images using MRIcroGL12.^56^ DWI images were used to guide lesion drawing as needed to ensure lesion boundaries were precisely demarcated.

### Calculating brain age

Each individual’s brain scan was ‘healed’ to exclude the effects of the stroke lesion on automated brain age estimates. First, each participant’s FLAIR/lesion maps were co-registered to align to their own T_1_ scan. Next, each participant’s T_1_ and spatially aligned lesion map were used to create an enantiomorphically healed version of their T_1_.^30^ The enantiomorphic healing process exploits the symmetrical nature of the brain (i.e. the right and left sides of the brain are roughly symmetrical), as well as the fact that the lesions in our sample were unilateral (and thus all lesions had corresponding contralateral intact brain tissue with which we could repair them). In the current study, enantiomorphic healing involved replacement of damaged tissue in the ipsilesional hemisphere with healthy tissue from homologous areas of the contralateral, non-lesioned hemisphere. This step was completed using the clinical toolbox.^57^ The enantiomorphically ‘healed’ brain image represents the best estimation of the structural integrity of the brain prior to stroke. All images were subject to visual inspection by study staff blinded to participants’ age. Because the BrainAgeR analysis pipeline expects images in native space as input, we did not normalize the enantiomorphically healed brains.

The BrainAgeR analysis pipeline (github.com/james-cole/brainageR)^24^ was applied to estimate biological brain age using default settings. First, the DARTEL toolbox^58^ in SPM12 was used to segment and normalize the T1 images. For quality control, probabilistic tissue maps were visually inspected by an expert neurologist to ensure proper segmentation. Second, cerebrospinal fluid was parcellated out, and grey and white matter probabilistic tissues were vectorized, concatenated, and fed into a principal component analysis (PCA) to reduce dimensionality. The PCA-derived components accounting for the top 80% of variance were retained for estimation of brain age. A pretrained Gaussian regression model in the R package Kernlab was implemented to predict brain age for each individual. The pretrained model was created based on input images from healthy individuals (*N* = 3377) and validated in a separate sample of healthy individuals (*N* = 611) between 18 and 90 years old,^24^ thus serving as inherent control data in the current study.

To adjust for variability in chronological age, we determined the proportional deviation of predicted brain age from chronological age as follows:

[(brainage−chronologicalage)/chronologicalage]

Each participant’s *proportional brain age difference* (PBAD) score indicates whether predicted age is accelerated or decelerated relative to her/his own chronological age. More specifically, positive values indicate premature brain ageing, whereas negative values suggest greater tissue integrity than the same age group in a normative sample.

### Statistical analyses

Multiple linear regression models were constructed to test the hypothesis that brain age at stroke onset is independently associated with language function (onset models). Each model included three terms: lesion volume, chronological age, and PBAD. Separate models were run for four outcome variables: overall score, naming, speech repetition, and auditory comprehension subscores. To test our second hypothesis, that brain age is associated with longitudinal recovery of language function, we applied the same paradigm for change in each subscore from stroke onset to follow-up assessment (recovery models). In addition to lesion volume, chronological age, and PBAD, the recovery models were adjusted for baseline performance on each language task (i.e. naming recovery model was adjusted for BEST-2 naming score at baseline). Associations between other variables were explored using Pearson’s or Spearman’s correlation coefficients as appropriate. All analyses were conducted in SPSS version 28.^59^

### Data availability

Data presented in this study is not publicly available at present. However, de-identifiable participant data is available from the primary author upon reasonable request.

## Results

**Figure 2:**
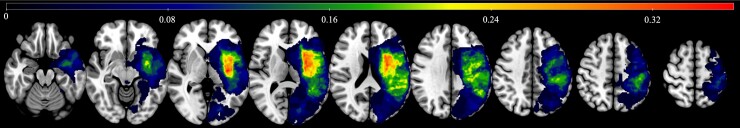
**Lesion overlay map.** Lesion overlap across participants. The colour bar represents proportional overlap (max = 37% overlap).

Fig. 2 presents a lesion overlay map for the study sample. Most participants presented with relatively small lesions (average lesion volume = 5.5 ± 6.0 cm^3^). Across the group, lesions primarily covered the middle cerebral artery peri-Sylvian region, with greatest overlap observed in the insula extending into inferior frontal territory.

**Figure 3:**
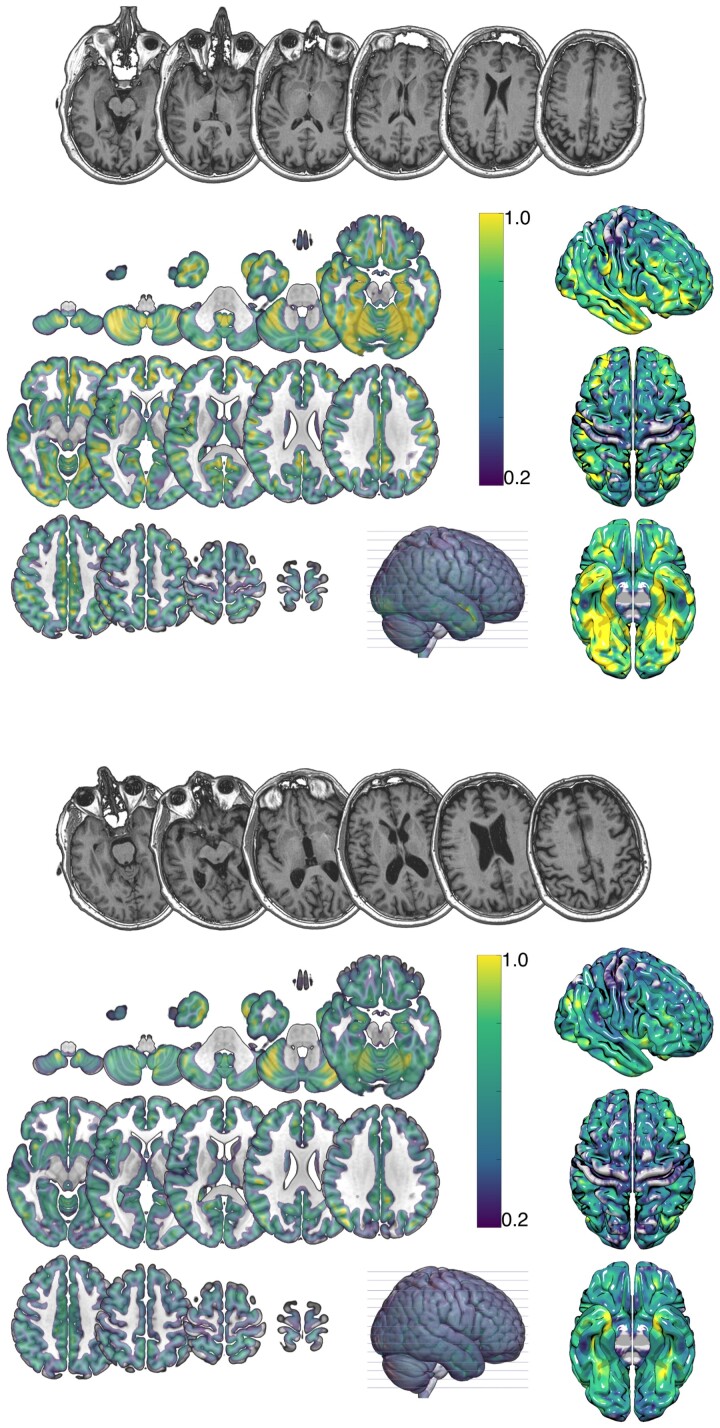
**Example grey matter volume maps.** Probabilistic grey matter estimates from two representative participants. Top panel: male, chronological age = 60.2 years, brain age = 36.9 years, PBAG = −0.39); bottom panel: male, chronological age = 62.4 years, brain age = 71.7, PBAG = 0.15). The colour bar represents the probabilistic measure of grey matter volume (darker colours suggesting less grey matter).

Brain age at stroke onset was estimated for all 49 participants. Estimated brain age was on average decelerated by 3.7 ± 7.5 years (range: −24.1 to 10.1 years) relative to chronological age. The corresponding PBAD values were −0.06 ± 0.11 (range: −0.40 to 0.16). Fig. 3 demonstrates an example of two participants of similar chronological age and with comparable lesion profiles but vastly different brain age.

Estimated brain age correlated significantly with chronological age (ρ = 0.80, *P* < 0.001) and with lesion volume (ρ = −0.29, *P* = 0.042). Chronological age was similarly correlated with lesion volume (ρ = −0.32, *P* = 0.026). Critically, PBAD was neither correlated with chronological age (ρ = −0.06, *P* = 0.704) nor with lesion volume (ρ = −0.07, *P* = 0.631).

### Brain age is associated with language function at stroke onset

**Figure 4:**
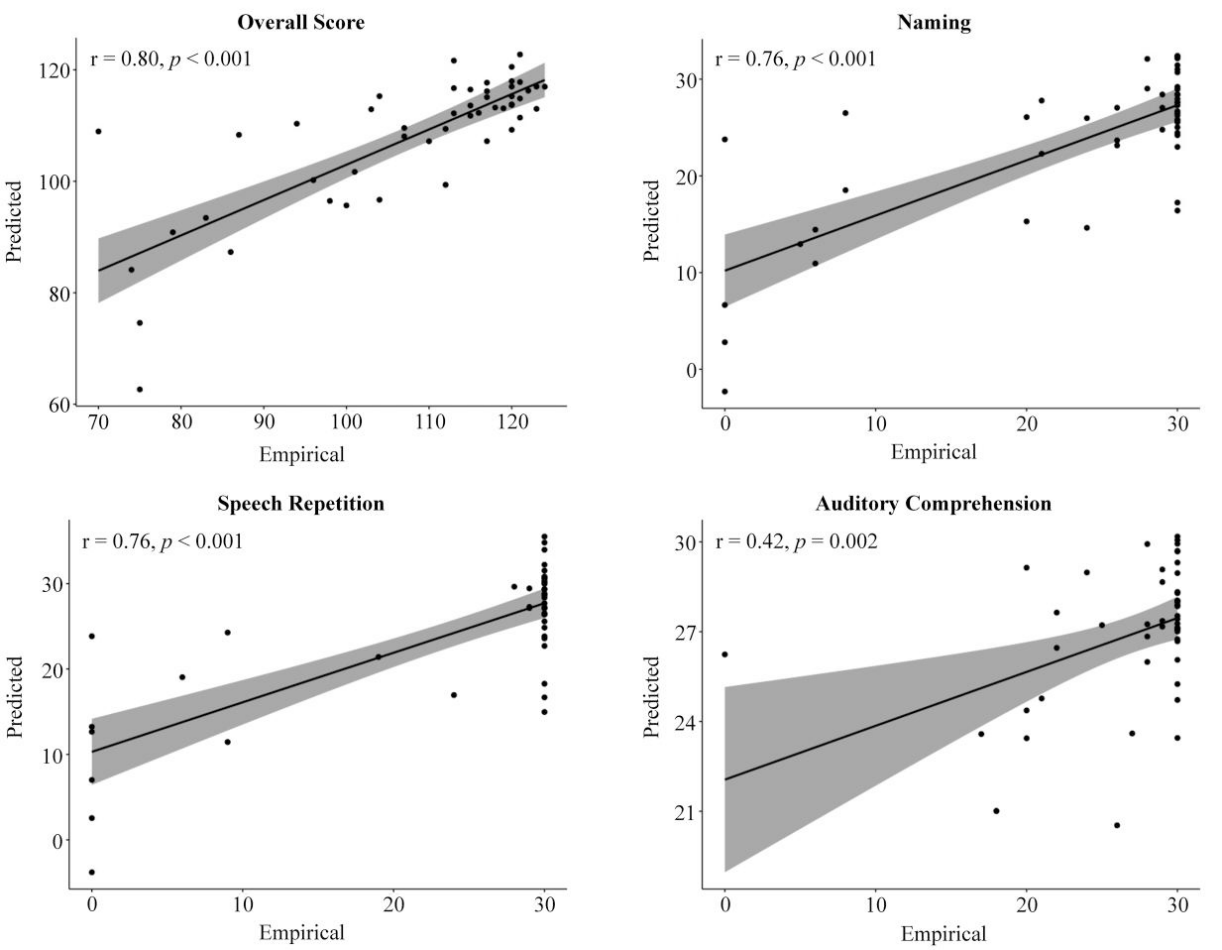
**Actual and predicted language scores.** Empirical versus predicted language scores at stroke onset. Multiple regression models included lesion volume, chronological age and brain age as independent terms.

Our first aim sought to test the hypothesis that estimated brain age is associated with language function at stroke onset independently of chronological age. To this end, multiple regression models were used to predict language outcomes based on lesion volume, chronological age, and PBAD. We found that PBAD was a significant predictor of overall score (F(1, 48) = 5.65, *P* = 0.022), naming (F(1, 48) = 5.13, *P* = 0.028), and speech repetition (F(1, 48) = 8.49, *P* = 0.006), but not auditory comprehension (F(1, 48) = 2.06, *P* = 0.158). In each case, the standardized beta (ß) value for PBAD was negative (−0.29 to −0.22; Table 1), suggesting a negative association between accelerated brain age relative to chronological age and language performance. Lesion volume was a significant predictor of all language outcomes (*P* = <0.001 to.005). Chronological age emerged as a significant predictor of naming (F(1, 48) = 4.18, *P* = 0.047), but the effect of chronological age was not significant in other models (all *P* > 0.20). Model parameters are shown in Table 1. Figure 4 demonstrates actual and predicted language scores based on onset models.

**Table 1:** Multiple regression models (df = 48) predicting language performance at stroke onset

	F	t	ß	η^2^	*P*
**Overall score**					
Model	25.97			0.63 (R^2^ = 0.61)	<0.001**
Lesion volume	72.72	−8.53	−0.82	0.62	<0.001**
Chronological age	0.41	−0.64	−0.06	0.01	0.525
PBAD	5.65	2.38	−0.22	0.11	0.022*
**Naming**					
Model	19.96			0.57 (R^2^ = 0.54)	<0.001**
Lesion volume	58.99	−7.68	−0.80	0.57	<0.001**
Chronological age	4.18	−2.05	−0.21	0.09	0.047*
PBAD	5.13	2.26	−0.23	0.10	0.028*
**Speech repetition**					
Model	20.70			0.58 (R^2^ = 0.55)	<0.001**
Lesion volume	57.91	−7.61	−0.79	0.56	<0.001**
Chronological age	1.18	−1.08	−0.11	0.03	0.284
PBAD	8.49	2.91	−0.29	0.16	0.006**
**Auditory comprehension**					
Model	3.29			0.18 (R^2^ = 0.13)	0.029
Lesion volume	8.93	−2.99	−0.43	0.17	0.005**
Chronological age	1.68	−1.30	−0.18	0.04	0.202
PBAD	2.06	1.44	−0.20	0.04	0.158

* ***P* < 0.05.**

** ***P* < 0.01.**

### Brain age is associated with longitudinal language recovery

**Figure 5:**
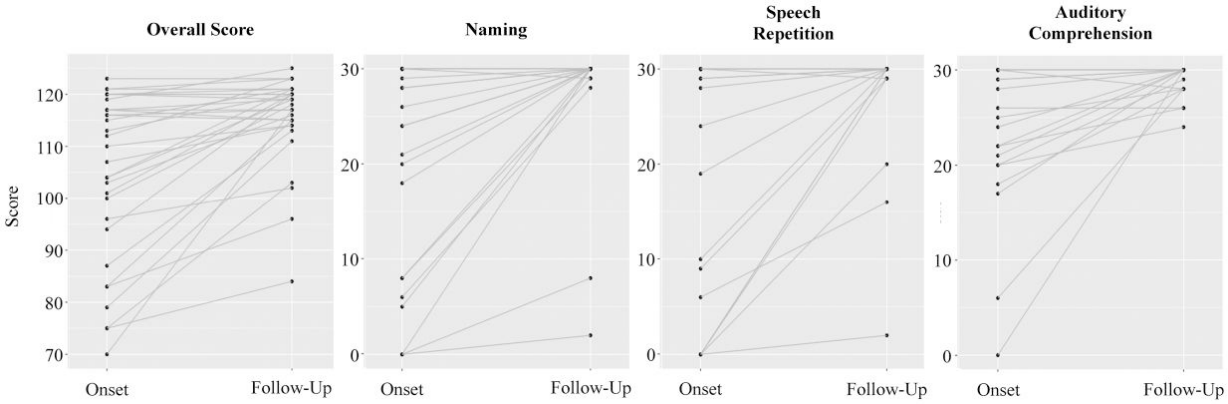
**Longitudinal recovery of language.** Longitudinal recovery across language domains. A paired-samples *t*-test revealed a significant average improvement on all language outcomes from stroke onset to follow-up (df = 29, t_overall_ = 4.8, t_naming_ = 3.4, t_repetition_ = 3.0, t_comprehension_ = 2.8, all *P* < 0.01).

On average, the subsample of participants who returned for a second language assessment showed a significant improvement on all language outcomes from stroke onset to follow-up (paired *t*(29) range: 2.8–4.8, all *P* < 0.01; Fig. 5). In order to examine the effects of brain age on longitudinal recovery of language function, we applied the same paradigm to model change in language performance from stroke onset to follow-up assessments (recovery models). Given the strong correlation between baseline and follow-up language assessments (*r* = 0.45–0.67, all *P* < 0.05), each recovery model was additionally adjusted for baseline BEST scores.

PBAD emerged as a significant predictor of change in overall language function from stroke onset to follow-up (F(1, 26) = 5.45, *P* = 0.028). The standardized beta coefficient was negative (ß = −0.22), suggesting that decelerated brain age relative to chronological age (negative PBAD value) was associated with better language recovery, whereas relatively accelerated brain age (positive PBAD value) was associated with poorer recovery. No statistically significant main effects of PBAD were observed for change in naming (F(1, 26) = 0.23, *P* = 0.880) or speech repetition (F(1, 26) = 0.11, *P* = 0.978), and PBAD marginally failed to reach statistical significance for change in auditory comprehension (F(1, 26) = 2.87, *P* = 0.103).

As expected, baseline performance was the strongest predictor in each model (all *P* < 0.001), independently accounting for > 70% of variability in language recovery. Lesion volume emerged as a significant predictor of change in overall score (*P* = 0.003), naming (*P* < 0.001), and auditory comprehension (*P* = 0.005) but marginally failed to reach statistical significance for speech repetition (*P* = 0.064). Chronological age was not associated with longitudinal change in any recovery model (all *P* > 0.20).

Given the variability in TPO at the follow-up assessment, we performed a *post hoc* analysis to investigate the effects of TPO on language recovery. TPO did not emerge as a significant factor in any model (all *P* > 0.30) and did not impact other results. Full models are shown in Table 2.

**Table 2:** General linear models predicting raw change in language performance from stroke onset to follow-up adjusted for baseline severity (BEST)

	F	t	ß	η^2^	*P*
**Overall score**					
Model	30.06			0.83 (R^2^ = 0.80)	<0.001
Baseline score	90.66	−9.52	−1.19	0.78	<0.001
Lesion volume	10.52	−3.24	−0.44	0.30	0.003
Chronological age	1.66	1.29	0.12	0.06	0.210
PBAD	5.45	−2.34	−0.22	0.18	0.028*
**Naming**					
Model	36.63			0.85 (R^2^ = 0.83)	<0.001
Baseline score	114.15	−10.68	−1.14	0.82	<0.001
Lesion volume	18.91	−4.35	−0.51	0.43	<0.001
Chronological age	0.64	0.80	0.07	0.03	0.433
PBAD	0.23	0.15	0.01	0.00	0.880
**Speech repetition**					
Model	28.77			0.82 (R^2^ = 0.79)	<0.001
Baseline score	69.13	−8.31	−1.05	0.73	<0.001
Lesion volume	3.77	−1.94	−0.26	0.13	0.064
Chronological age	0.69	0.83	0.08	0.03	0.413
PBAD	0.00	0.03	0.00	0.00	0.978
**Auditory comprehension**					
Model	159.39			0.96 (R^2^ = 0.96)	<0.001
Baseline score	547.88	−23.41	−1.03	0.96	<0.001
Lesion volume	9.56	−3.09	−0.15	0.28	0.005
Chronological age	0.58	−0.76	−0.03	0.02	0.453
PBAD	2.87	−1.69	−0.07	0.10	0.103

* *P* < 0.05.

Finally, we ran each recovery model without adjustment for baseline BEST scores to enable direct comparison to the cross-sectional results reported above. Together, lesion volume, chronological age, and PBAD accounted for 19%, 33%, 14%, and 20% of variability in change scores in naming, speech repetition, auditory comprehension, and overall score, respectively. Without adjustment for baseline performance, PBAD was associated with change in speech repetition (*P* = 0.017) and marginally failed to reach statistical significance for change in naming (*P* = 0.072), whereas chronological age was not a significant factor in any model (all *P* > 0.100; Supplementary Table 1).

## Discussion

This study tested the hypothesis that brain age, as estimated based on neuroimaging-derived measures of brain atrophy, is associated with language function and recovery following stroke independent of chronological age. Our results support this hypothesis. Specifically, we found that accelerated brain age relative to chronological age is negatively associated with both language function at stroke onset and long-term language recovery. This effect was independent of overall lesion volume and TPO. Thus, the present study demonstrates for the first time the utility of brain age estimated based on routine clinical-grade brain scans to inform longitudinal recovery of language function following left hemisphere stroke. The significance of these findings is discussed below.

### Association between age and language performance in stroke

Neuroplastic properties of the brain deteriorate with age due to progressive atrophy of grey and white matter tissue.^60,61^ As a consequence, healthy ageing is accompanied by gradual cognitive decline,^62,63^ including in language function.^64^ The rate of age-related cognitive decline is associated with increased risk of neurogenic diseases, such as dementia.^62^ Moreover, the diminished structural integrity of the brain has been shown to be associated with worse functional outcomes in stroke recovery.^65,66^

Despite ample evidence suggesting a strong causal link between structural integrity of intact brain regions and recovery, prior work has failed to find a consistent relationship between age and language recovery in post-stroke aphasia.^9^ Several potential reasons for this contradiction have been postulated. For instance, some studies have observed more severe language deficits in older patients at stroke onset.^1,2^ As aphasia severity is generally considered a strong predictor of language recovery,^4,5^ this may negate any possible independent effects of age. Alternatively, the large interindividual variability in age-related brain changes^22,67^ may reduce statistical power to detect effects of interest in a literature that is dominated by single-subject and small group studies.^68^

Predicted brain age largely bypasses these issues and offers a novel approach to inform the true integrity of the brain.^44^ Our results revealed a positive correlation between chronological age and brain age (ρ = 0.80, *P* < 0.001), suggesting that these two measures are strongly related. Notwithstanding, we found that the relative deviance between estimated brain age and age was associated with performance on naming and speech repetition, as well as overall score at stroke onset (see Table 1) and longitudinal recovery of overall language function (see Table 2) when variability explained by chronological age was accounted for. In further *post hoc* analysis, all significant main effects were replicated after ceiling scores were removed (Supplementary Tables 2 and 3). These findings are consistent with the notion that there is not a direct correspondence between chronological age and cognitive decline^69,70^ and, instead, indicate that estimated brain age accounts for unique variability unrelated to chronological age.

Recent research has shown that other cerebrovascular risk factors are similarly correlated with brain age, such as blood pressure^71^ and BMI.^13^ Cerebrovascular biomarkers are unequivocally associated with overall brain health and structural brain atrophy.^72,73^ To this end, estimated brain age may capture atrophy explained by other factors than chronological age. In the context of the current study, these additional factors account for a significant amount of variability in language function and recovery. Importantly, our results echo findings in other neurogenic diseases^74–76^ and corroborate recent findings reported in the stroke recovery literature.^42,45,46^

### Implications

While prior studies in the aphasia literature have not incorporated an estimate of brain age to inform language function, various approaches have been successfully implemented to reveal a strong association between structural integrity of intact brain regions and language performance.^77,78^ The novelty of the current study lies instead in the approach used. We applied enantiomorphic ‘healing’ to clinical T_1_-weighted brain scans to avoid complications introduced by lesioned brain tissue and enable accurate computation of brain age. Proportional brain age gap was unrelated to lesion volume, indicating that the healed brain image was unaffected by lesion characteristics. This is important for two main reasons. First, the sheer extent of lesion damage is a critical determinant of the subsequent functional consequences.^7^ This notion is strongly supported by our findings as lesion volume was a strong predictor in most regression models, typically accounting for one- to two-thirds of variability in the dependent variable. Critically, the effect of brain age was independent of lesion volume.

Second, measures of structural integrity used to investigate language function in post-stroke aphasia are frequently derived from DWI, T_2_-weighted scans, or other sophisticated imaging modalities that use long acquisition times, multi-echo sequences, and ultra-high field resolution only possible on high field strength (3 T) scanners. These research-grade scans are generally not collected as part of routine clinical care in stroke, where the primary goal is to acquire time-sensitive information about coarse lesion characteristics. In the current study, the scans came from a 1.5 T scanner, which is common for clinical scans. The ability to derive clinically meaningful prognostic information from clinical scans offers the potential to substantially improve prognostication procedures in aphasia.^79^

Therefore, the current study serves as a proof-of-principle for a novel, effective and simple to use approach to inform post-stroke language recovery. The extent to which brain age, as indicative of total and/or regional brain atrophy, can be implemented as a tool to guide clinical decision making in aphasia remains to be examined. Future studies will need to determine the unique contribution of brain age relative to other lesion, neuropsychological, and biographical factors associated with language outcomes. As a biomarker of cognitive reserve, brain age is less dependent on factors like language, education, and socio-economic status, which frequently influence cognitive testing.^80,81^ At the same time, brain age is sensitive both to modifiable environmental factors, such as training,^28,39^ and changes in cognitive abilities.^82,83^ Thus, brain age may be a particularly promising marker of long-term therapy success.

### Limitations

The results reported herein, despite being promising, should be interpreted with caution given the novel approach implemented. Several other important limitations of the study design warrant discussion. First, and perhaps most importantly, we included a relatively small sample size that may not support generalization of the results to another sample. Although the sample size is fairly typical for aphasia research,^84^ the heterogenous nature of language deficits in aphasia reduces statistical power to detect subtle effects of interest.^85^ Notwithstanding, it is worth noting that the strength of the association between brain age and language performance in the current study increases our confidence that these findings are not spurious.

Second, estimated brain age was considerably lower on average than chronological age (mean = −3.7 years). This estimate is lower than that reported in most prior studies.^86^ There are several potential reasons for this; one potential reason is that cerebrovascular health statistics are generally good in Iceland, especially for women.^87^ Importantly, 25/49 participants in the current study were women. Additionally, the Icelandic population has comparatively good access to high quality health care at a low out-of-pocket cost.^88^

Third, one potential criticism of this work is that the enantiomorphic healing process could have introduced artefacts into the brain images that were then used by BrainAgeR to estimate age. We argue that this is unlikely due to our finding that lesion size (and thus the extent to which damaged tissue was replaced with healthy tissue) was not significantly related to estimated brain age differences. Last, the BEST-2 is a coarse measure that may not be sensitive to subtle changes in language function. However, given the substantial functional changes expected in the acute recovery phase^89^ in addition to observed improvements across all language tests, this should not affect our results.

## Conclusions

In conclusion, our results show for the first time that neuroimaging-based estimation of brain age—as a measure of overall structural integrity of the brain—is associated with language function and recovery following acute stroke. Critically, brain age explained more variability in language performance than chronological age alone. These results hold substantial promise to enhance understanding of the neural bases of aphasia recovery and to improve prognostication in the clinical management of aphasia.

## Supplementary Material

fcac252_Supplementary_Data

## Abbreviations

BEST-2: Bedside Evaluation Screening Test-Second Edition
DWI: diffusion-weighted imaging
FLAIR: fluid-attenuated inversion recovery
PBAD: proportional brain age difference
PCA: principal component analysis
SD: standard deviation
TE: echo time
TI: inversion time
TPO: time post-onset
TR: repetition time.
