# Supplementary material for: Brain age predicts long-term recovery in post-stroke aphasia
Source: Brain Commun. 2022 Oct 6;4(5):fcac252. doi: 10.1093/braincomms/fcac252 (PMC9576153; doi:10.1093/braincomms/fcac252)
Supplement: fcac252_Supplementary_Data [file fcac252_supplementary_data.zip › Original Submission.pdf]

OXFORD UNIVERSITY PRESS | Brain Communications

Brain Age Predicts Long-Term Recovery in Post-Stroke Aphasia

Journal:	*Brain Communications*
Manuscript ID	BRAINCOM-2022-082
Manuscript Type:	Original Article
Date Submitted by the Author:	23-Feb-2022
Complete List of Authors:	Kristinsson, Sigfus; University of South Carolina, Communication Sciences and Disorders<br>Busby, Natalie; University of South Carolina, Department of Communication Sciences and Disorders<br>Rorden, Christopher; University of South Carolina, McCausland Center for Brain Imaging, Psychology<br>Newman-Norlund, Roger; University of South Carolina, Department of Communication Sciences and Disorders<br>den Ouden, Dirk; University of South Carolina, Communication Sciences and Disorders<br>Magnusdottir, Sigridur; University of Iceland<br>Hjaltason, Haukur; University of Iceland<br>Thors, Helga; University of Iceland<br>Hillis, Argye; Johns Hopkins University, Neurology<br>Bonilha, Leonardo; University of South Carolina, Neuropsychiatry and Communication Disorders and Sciences<br>Fridriksson, Julius ; University of South Carolina, Communication Sciences and Disorders
Keywords:	

SCHOLARONE™
Manuscripts

Brain Age Predicts Long-Term Recovery in Post-Stroke Aphasia

Running Head: Brain Age in Aphasia

For Review Only

Sigfus Kristinsson, PhD[1*], Natalie Busby, PhD[1], Chris Rorden, PhD[1,2], Roger Newman-Norlund, PhD[1,2], Dirk B. den Ouden, PhD[1,3], Sigridur Magnusdottir, PhD[4], Haukur Hjaltason, MD, PhD[4,5], Helga Thors, PhD[4], Argye E. Hillis, MD, PhD[1,6], Olafur Kjartansson, MD[5], Leonardo Bonilha, MD, PhD[1,7‡], & Julius Fridriksson, PhD, CCC-SLP[1,3‡]

[1]Center for the Study of Aphasia Recovery, University of South Carolina, Columbia, SC, USA
[2]Department of Psychology, University of South Carolina, Columbia, SC, USA
[3]Department of Communication Sciences and Disorders, Columbia, SC, USA
[4]Department of Medicine, University of Iceland
[5]Department of Neurology, Landspitali University Hospital
[6]Department of Neurology, Johns Hopkins University School of Medicine
[7]Department of Neurology, Medical University of South Carolina, Charleston, SC, USA

‡ Shared final authorship.

*Corresponding Author
Sigfus Kristinsson, PhD
Center for the Study of Aphasia Recovery
University of South Carolina
915 Greene Street
Columbia, SC 29209
803-553-4689
sigfus@email.sc.edu
Twitter handle: @SigfusHelgi

Keywords: Aphasia, Neuroimaging, Brain Age, Age

Abstract

Background: The association between age and language recovery in stroke remains unclear. Here, we examined the association between neuroimaging-based brain age, a measure of structural integrity, at stroke onset and: (1) cross-sectional language function, and (2) long-term recovery of language function, beyond chronological age.

Method: A total of 49 participants (age: 65.2 +/- 12.2 years, 25 female) underwent routine clinical neuroimaging (T1) and a bedside evaluation of language performance (Bedside Evaluation Screening Test-2) at onset of left hemisphere stroke. Brain age was estimated from enantiomorphically reconstructed brain scans using a machine learning algorithm trained on a large sample of healthy adults. A subsample of 30 participants returned for follow-up language assessments at least two years after stroke onset. Multiple regression models were constructed to test the effects of brain age on language outcomes. Lesion volume and chronological age were included as covariates in all models.

Results: Higher brain age was associated with worse overall aphasia severity ($F(1, 48) = 5.65$, $p = .022$), naming ($F(1, 48) = 5.13$, $p = .028$), and speech repetition ($F(1, 48) = 8.49$, $p = .006$) at stroke onset. Follow-up assessments were carried out ≥2 years after onset; brain age was found to be inversely associated with change in aphasia severity ($F(1, 26) = 8.66$, $p = .007$) and speech repetition ($F(1, 26) = 7.10$, $p = .013$), but its correlation with change in naming ($F(1, 26) = 3.4$, $p = .078$) and auditory comprehension ($F(1, 26) = 3.3$ $p = .081$) marginally failed to reach statistical significance. Chronological age was only associated with naming performance at stroke onset ($F(1, 48) = 4.18$, $p = .047$).

Conclusion: These results indicate that brain age as estimated based on routine clinical brain scans may be a strong biomarker for language function and recovery after stroke.

For Review Only

Introduction

Aphasia is a language impairment that is generally recognized as one of the most disabling consequences of a stroke affecting the language-dominant brain hemisphere.[1] Most individuals with aphasia recover some language functions in the days and months following the stroke[2], but the factors associated with recovery remain poorly understood.[3] Prior studies indicate that the initial severity of aphasia[4,5], the size of the cortical infarct[6,7], and the lesion site[6,8] account for substantial variability in long-term outcomes.

The relationship between recovery and other variables such as age is less clear.[9] Neuroplastic properties of the brain decrease with age[10,11], suggesting that age might be an important factor in aphasia recovery. However, older individuals are more likely to present with severe aphasia[1,2], which may negate the direct association between age and recovery. Recent research suggests that brain age, which is based on an estimate of cortical tissue integrity, is a more useful indicator of neuroplastic properties of the brain.[12,13] In the current study, we report the first acute-to-chronic examination of the impact of estimated brain age for longitudinal language recovery in aphasia.

Healthy ageing is accompanied by reliable changes to structural integrity of the brain; in particular, atrophy of grey matter, reduced volume of white matter connections, and distorted functional connectivity have been observed with magnetic resonance imaging.[14-22] The recently coined concept of *brain age* broadly represents these changes.

Brain age is generally predicted using machine learning algorithms that leverage neuroimaging-derived measures of structural atrophy to estimate how old the brain looks compared to a large sample of healthy control subjects.[12] The extent to which brain age deviates from chronological age has been found to be associated with onset of psychiatric and neurologic diseases[13,23], physical functioning[24,25], and cognitive abilities.[24,26-28] This suggests that estimated brain age may potentially be implemented as a surrogate measure for cognitive reserve.

The presence of a brain lesion presents a challenge for the estimation of brain age since current approaches depend on the quality of normalization of the neuroimages, i.e., warping individual brains into standard space. Necrotic brain tissue can markedly distort the normalization, which is generally designed to process healthy brain images.[29] This issue can be bypassed by applying an enantiomorphic algorithm to native T1 images to effectively 'heal' the damaged hemisphere.[30] The enantiomorphic 'healing' takes advantage of the left-right symmetry across hemispheres to replace tissue in the damaged hemisphere with a mirror image of healthy tissue from the contralateral hemisphere. This approach has been successfully applied in our prior work[31-34] as well as by other groups.[35-37]

The rate of brain atrophy is increasingly implemented as a clinical biomarker in various neurological disorders characterized by a marked deviation between brain age and chronological age.[38-40] In the context of stroke, recent studies have emphasized the detrimental impact of stroke as manifested in accelerated brain age.[41,42] Others have observed an association between brain age and stroke risk[43], potentially indicating that biological brain age may both be a biomarker for stroke risk and exacerbated as a consequence of brain damage. Critically, while the association between neurodegeneration and cognitive function has been observed in many neurological

For Review Only

disorders[23,26,44], the relationship between brain age and cognitive outcomes in post-stroke functional recovery remains to be studied in detail.[45-48]

To this end, we examined the association between brain age and language outcomes after stroke. Specifically, we tested the hypothesis that brain age at stroke onset is associated with: (1) cross-sectional language function and (2) long-term recovery of language function, *beyond chronological age*. We expected accelerated brain age to be associated with poorer language function and worse recovery. This study leveraged retrospective high-quality neuroimaging data and language assessments collected at stroke onset and at least two years after stroke onset.

For Review Only

Methods

Participants

A total of 49 individuals with acute left hemisphere injury were included in the study. Participants were recruited through the neurology ward at the National University Hospital of Iceland, Reykjavk. Participants were eligible for inclusion in the study if they (1) had incurred a single, unilateral left hemisphere stroke, (2) were in the acute phase of recovery, (3) had their stroke confirmed by a CT/MRI scan, (4) had no history of major psychiatric illness or other neurologic impairment affecting the brain, (5) were native speakers of Icelandic, and (6) gave informed consent for study participation. All study procedures were approved by the Institutional Review Board of the University of Iceland. For a detailed description of participants and procedures, see Magnusdottir et al. (2013, ref. 49) and Kristinsson et al. (2020, ref. 50).

Participants underwent MRI imaging and language assessments within three days of hospital admission. At stroke onset, the average age of the sample was 65.2 years (SD = 12.2 years, range: 34-85 years) and 25 participants were female. A subsample of 30 participants returned for a follow-up language assessment at least 24 months post-onset. At the time of retesting, the average age of the sample was 67.5 years (SD = 10.2 years, range: 43-82 years). The range of time post stroke across participants was 2.4-5.4 years (mean = 4.0 years, SD = 0.9 years) at retesting. Figure 1 shows TPO across participants.

[Insert Figure 1]

Language Assessments

Speech and language impairment was assessed with the Bedside Evaluation Screening Test-Second Edition (BEST-2).[51] The BEST-2 is designed to assess language function at bedside in acute patients who may not be able to complete a full language assessment battery. In addition to providing an assessment of overall language impairment (henceforth, overall score), the BEST-2 assesses several language domains, including naming, speech repetition, and auditory comprehension. As these domains correspond to the main subtests on the Western Aphasia Battery-Revised (WAB-R)[52], which is the most widely used aphasia test[53], we included all four scores in the data analyses. Importantly, despite being a short evaluation, our prior work has shown that the BEST-2 is sensitive to language impairment and longitudinal changes in language function.[49,50,54]

Magnetic Resonance Imaging

MRI data were collected as part of routine clinical care in acute stroke on a 1.5T Siemens scanner. We obtained T1-weighted images, diffusion-weighted images (DWI), and fluid-attenuated inversion recovery (FLAIR) scans. The details for these sequences are as follows: *T1-weighted image* (3D GR\IR sequence, TR = 1,160 ms, TI = 600 ms, TE = 4.24 ms, flip angle = 15°, the 256 x 256 matrix was reconstructed at 512 x 512, yielding a 0.45 x 0.45 mm^2 in axial-plane resolution, with 192 0.9 mm slices), *diffusion-weighted images* (three scans with B0 = 0, 500, and 1,000; TR = 3,808 ms, TE = 89 ms, flip angle = 90°, Nx = 4, 192 x 192 matrix, 1.2 x 1.2 mm^2 in axial plane, 24 slices, each 5 mm thick with 1.5-mm gap), and *T2-weighted FLAIR image* (TR = 9,000 ms, TI = 2,500 ms, TE = 112 ms, flip angle = 15°, 280 x 320 matrix with

For Review Only

0.72 x 0.72 mm^2 in axial plane resolution, 24 slices, each 5 mm thick with 1.5-mm gap). Images were converted from DICOM to NIfTI format using dcm2niix[55], which preserves spatial coordinates (yielding a good starting estimate for the subsequent co-registration of the T1 image to the T2 scan).

An expert neurologist or trained study staff member with extensive experience/training in lesion drawing manually demarcated the brain lesions on FLAIR images using MRIcroGL12.[56]

Calculating Brain Age

Each individual's brain scan was 'healed' to exclude the effects of the stroke lesion on automated brain age estimates. First, each participant's FLAIR/lesion maps were co-registered to align to their own T1 scan. Next, each participant's T1 and spatially-aligned lesion map were used to create an enantiomorphically healed version of their T1.[30] The enantiomorphic healing process exploits the symmetrical nature of the brain (i.e., the right and left sides of the brain are roughly symmetrical), as well as the fact that the lesions in our sample were unilateral (and thus all lesions had corresponding contralateral intact brain with which we could repair them). In the current study, enantiomorphic healing involved replacement of damaged tissue in the ipsilesional hemisphere with healthy tissue from homologous areas of the contralateral, non-lesioned hemisphere. This step was completed using the clinical toolbox.[57] The enantiomorphically 'healed' brain image represents the best estimation of the structural integrity of the brain prior to stroke. All images were subject to visual inspection by study staff blinded to participants' age. Because the BrainAgeR analysis pipeline expects images in native space as input, we did not normalize the enantiomorphically healed brains.

The BrainAgeR analysis pipeline (github.com/james-cole/brainageR)[24] was applied to estimate biological brain age using default settings. First, the DARTEL toolbox[58] in SPM12 was used to segment and normalize the T1 images. For quality control, probabilistic tissue maps were visually inspected by an expert neurologist to ensure proper segmentation. Second, cerebrospinal fluid was parcellated out, and gray and white matter probabilistic tissues were vectorized, concatenated, and fed into a principal component analysis (PCA) to reduce dimensionality. The PCA-derived components accounting for the top 80% of variance were retained for estimation of brain age. A pretrained Gaussian regression model in the R package Kernlab was implemented to predict brain age for each individual. The pretrained model was created based on input images from healthy individuals (N = 3,377) and validated in a separate sample of healthy individuals (N = 611) between 18 and 90 years old[24], thus serving as inherent control data in the current study. Figure 3 demonstrates a visual representation of the brain age estimation.

To adjust for variability in chronological age, we determined the proportional deviation of predicted brain age from chronological age as follows:

$$[(\text{brain age} - \text{chronological age}) / \text{chronological age}]$$

Each participant's proportional brain age difference (PBAD) score indicates whether predicted age is accelerated or decelerated relative to her/his own chronological age. More specifically, positive values indicate premature brain aging, whereas negative values suggest greater tissue integrity than the same age group in a normative sample.

For Review Only

Statistical Analyses

Multiple linear regression models were constructed to test the hypothesis that brain age at stroke onset is independently associated with language function. Each model included three terms: lesion volume, chronological age, and brain age (PBAD). Separate models were run for four outcome variables: overall score, naming, speech repetition, and auditory comprehension subscores. To test our second hypothesis, that brain age is associated with longitudinal recovery of language function, we applied the same paradigm in a longitudinal design. Repeated measures analyses of variance (ANOVA) with two time points (baseline and follow-up) were used to model language outcomes obtained at follow-up. Repeated ANOVAs were built for the same language tasks as before and included the same independent terms, in addition to adjustment for baseline language performance. Associations between other variables were explored using Pearson's or Spearman's correlation coefficients as appropriate. All analyses were conducted in SPSS version 28.[59]

Results

Figure 2 presents a lesion overlay map for the study sample. Most participants presented with relatively small lesions (average lesion volume = 5.5 +/- 6.0 cm^3). Across the group, lesions primarily covered the middle cerebral artery peri-Sylvian region, with greatest overlap observed in the insula extending into inferior frontal territory.

[Insert Figure 2]

Brain age at stroke onset was estimated for all 49 participants. Estimated brain age was on average decelerated by 3.7 +/- 7.5 years (range: -24.1 to 10.1 years) relative to chronological age. The corresponding proportional brain age difference values were -.06 +/- .11 (range: -.40 to .16). Figure 3 demonstrates an example of two participants of similar chronological age and with comparable lesion profiles, but vastly different brain age.

[Insert Figure 3]

Figure 1. Probabilistic gray matter estimates from two representative participants. Top panel: Male, chronological age = 60.2 years, brain age = 36.9 years, PBAG = -.39); bottom panel: Male, chronological age = 62.4 years, brain age = 71.7, PBAG = .15). The colorbar represents the probabilistic measure of gray matter volume (darker colors suggesting less gray matter).

Estimated brain age correlated significantly with chronological age ($\rho = .80$, $p < .001$) and with lesion volume ($\rho = -.29$, $p = .042$). Chronological age was similarly correlated with lesion volume ($\rho = -.32$, $p = .026$). Critically, PBAD was neither correlated with chronological age ($\rho = -.06$, $p = .704$) nor with lesion volume ($\rho = -.07$, $p = .631$).

For Review Only

Brain Age is Associated with Language Function at Stroke Onset

Our first aim sought to test the hypothesis that estimated brain age is associated with language function at stroke onset independently of chronological age. To this end, multiple regression models were used to predict language outcomes based on lesion volume, chronological age, and brain age (PBAD). We found that brain age was a significant predictor of overall score (F(1, 48) = 5.65, *p* = .022), naming (F(1, 48) = 5.13, *p* = .028), and speech repetition (F(1, 48) = 8.49, *p* = .006), but not auditory comprehension (F(1, 48) = 2.06, *p* = .158). Lesion volume was a significant predictor of all language outcomes (*p* = <.001 to .005). Chronological age emerged as a significant predictor of naming (F(1, 48) = 4.18, *p* = .047), but the effect of chronological age was not significant in other models (all *p* > .20). Model parameters are shown in Table 1. Figure 4 plots actual and predicted language scores across the whole group of participants and split into two age groups based on estimated brain age (50/50: older/younger).

	F	t	ß	η^2	*p*
			**Overall Score**		
Model	25.97			.63 (R^2 = .61)	<.001**
Lesion volume	72.72	-8.53	-.82	.62	<.001**
Chronological age	.41	-.64	-.06	.01	.525
Brain age	5.65	2.38	-.22	.11	.022*
			**Naming**		
Model	19.96			.57 (R^2 = .54)	<.001**
Lesion volume	58.99	-7.68	-.80	.57	<.001**
Chronological age	4.18	-2.05	-.21	.09	.047*
Brain age	5.13	2.26	-.23	.10	.028*
			**Speech Repetition**		
Model	20.70			.58 (R^2 = .55)	<.001**
Lesion volume	57.91	-7.61	-.79	.56	<.001**
Chronological age	1.18	-1.08	-.11	.03	.284
Brain age	8.49	2.91	-.29	.16	.006**
			**Auditory Comprehension**		
Model	3.29			.18 (R^2 = .13)	.029
Lesion volume	8.93	-2.99	-.43	.17	.005**
Chronological age	1.68	-1.30	-.18	.04	.202
Brain age	2.06	1.44	-.20	.04	.158

Table 1. Multiple regression models (df = 48) predicting language performance at stroke onset. *p < .05, **p < .01.

[Insert Figure 4]

Brain Age is Associated with Longitudinal Language Recovery

On average, the subsample of participants who returned for a second language assessment showed a significant improvement on all language outcomes from stroke onset to follow-up (all *p* < .001). In order to examine the effects of brain age on longitudinal recovery of language

For Review Only

function, we applied the same modeling paradigm in a repeated-measures design. Estimated brain age emerged as a significant predictor of change in overall language function ($F(1, 26) = 8.66$, $p = .007$) and speech repetition ($F(1, 26) = 7.1$, $p = .013$), and marginally failed to reach statistical significance as a predictor for naming ($F(1, 26) = 3.4$, $p = .078$) and auditory comprehension ($F(1, 26) = 3.3$, $p = .081$). Lesion volume was a significant predictor in all models (p = <.001 to .004), whereas chronological age was not a significant predictor in any model (all $p > .05$). Given the variability in time post-onset (TPO) at the follow-up assessment, we performed a *post hoc* analysis to investigate the effects of TPO in these models. TPO did not emerge as a significant factor in any model (all $p > .30$) and did not impact other results. Full models are shown in Table 2, and Figure 5 demonstrates individual recovery trajectories by brain age (younger/older).

	df	MS	F	η^2	*p*
			**Overall**		
Intercept	1	15,802.1	171.3	.87	<.001**
Lesion volume	1	4,404.3	47.7	.65	<.001**
Chronological age	1	.0	.0	.00	.986
Brain age	1	798.5	8.66	.25	.007**
Error	26	92.2			
			**Naming**		
Intercept	1	1,363.9	29.8	.53	<.001**
Lesion volume	1	2,134.2	46.7	.64	<.001**
Chronological age	1	22.4	.5	.02	.491
Brain age	1	154.2	3.4	.12	.078
Error	26	45.7			
			**Speech Repetition**		
Intercept	1	1,079.4	19.9	.43	<.001**
Lesion volume	1	1,919.2	35.3	.58	<.001**
Chronological age	1	1.7	.0	.00	.862
Brain age	1	384.5	7.1	.21	.013*
Error	26	54.4			
			**Auditory Comprehension**		
Intercept	1	1,475.5	77.0	.75	<.001**
Lesion volume	1	186.8	9.7	.27	.004**
Chronological age	1	61.9	3.2	.11	.084
Brain age	1	63.3	3.3	.11	.081
Error	26	19.2			

Table 2. Repeated-measures ANOVA predicting change in language performance from stroke onset to follow-up. MS = mean squares; *$p < .05$, **$p < .01$.

[Insert Figure 5]

For Review Only

Discussion

This study tested the hypothesis that brain age, as estimated based on neuroimaging-derived measures of brain atrophy, is associated with language function and recovery following stroke independent of chronological age. Our results support this hypothesis. Specifically, we found that accelerated brain age is negatively associated with both language function at stroke onset and long-term language recovery. This effect was independent of overall lesion volume and time post-onset. Thus, the present study demonstrates for the first time the utility of brain age estimated based on routine clinical-grade brain images to inform longitudinal recovery of language function in aphasia. The significance of these findings is discussed below.

Association Between Age and Language Performance in Stroke

Neuroplastic properties of the brain deteriorate with age due to progressive atrophy of gray and white matter tissue.[60,61] As a consequence, healthy ageing is accompanied by gradual cognitive decline[62,63], including in language function.[64] The rate of age-related cognitive decline is associated with increased risk of neurogenic diseases, such as dementia.[62] Moreover, the diminished structural integrity of the brain has been shown to be associated with worse functional outcomes in stroke recovery.[65,66]

Despite ample evidence suggesting a strong causal link between structural integrity of intact brain regions and recovery, prior work has failed to find a consistent relationship between age and language recovery in post-stroke aphasia.[9] Several potential reasons for this contradiction have been postulated. For instance, some studies have observed more severe language deficits in older patients at stroke onset.[1,2] As aphasia severity is generally considered a strong predictor of language recovery[4,5], this may negate any possible independent effects of age. Alternatively, the large interindividual variability in age-related brain changes[22,67] may reduce statistical power to detect effects of interest in a literature that is dominated by single-subject and small group studies.[68]

Predicted brain age largely bypasses these issues and offers a novel approach to inform the true integrity of the brain.[44] Our results revealed a positive correlation between chronological age and brain age ($\rho = .80$, $p < .001$), suggesting that these two measures are strongly related. Notwithstanding, we found that brain age was associated with both language function at stroke onset (see Table 1) and language recovery (see Table 2) in several language domains when variability explained by chronological age was accounted for. These findings are consistent with the notion that there is not a direct correspondence between chronological age and cognitive decline[69,70] and, instead, indicate that estimated brain age accounts for unique variability unrelated to chronological age.

Recent research has shown that other cerebrovascular risk factors are similarly correlated with brain age, such as blood pressure[71] and BMI.[13] Cerebrovascular biomarkers are unequivocally associated with overall brain health and structural brain atrophy.[72,73] To this end, estimated brain age may capture atrophy explained by other factors than chronological age. In the context of the current study, these additional factors account for a significant amount of variability in language function and recovery. Importantly, our results echo findings in other neurogenic diseases[74-76] and corroborate recent findings reported in the stroke recovery literature.[42,45,46]

Implications

While prior studies in the aphasia literature have not incorporated an estimate of brain age to inform language function, various approaches have been successfully implemented to reveal a strong association between structural integrity of intact brain regions and language performance.[77,78] The novelty of the current study lies instead in the approach used. We applied enantiomorphic 'healing' to clinical T1-weighted brain scans to avoid complications introduced by lesioned brain tissue and enable accurate computation of brain age. Proportional brain age gap was unrelated to lesion volume, indicating that the healed brain image was unaffected by lesion characteristics. This is important for two main reasons. First, the sheer extent of lesion damage is a critical determinant of the subsequent functional consequences.[7] This notion is strongly supported by our findings as lesion volume was by far the strongest predictor in each regression model, typically accounting for one- to two-thirds of variability in the dependent variable. Critically, the effect of brain age was independent of lesion volume.

Second, measures of structural integrity used to investigate language function in post-stroke aphasia are frequently derived from diffusion-weighted imaging (DWI), T2-weighted scans, or other sophisticated imaging modalities that use long acquisition times, multi-echo sequences, and ultra-high field resolution only possible on high field strength (3T) scanners. These research-grade scans are generally not collected as part of routine clinical care in stroke, where the primary goal is to acquire time-sensitive information about coarse lesion characteristics. In the current study, the scans came from a 1.5T scanner, which is common for clinical scans. The ability to derive clinically meaningful prognostic information from clinical scans offers the potential to substantially improve prognostication protocols in aphasia.[79]

For Review Only

Therefore, the current study serves as a proof-of-principle for a novel, effective, and simple to use approach to inform post-stroke language recovery. The extent to which brain age, as indicative of total and/or regional brain atrophy, can be implemented as a tool to guide clinical decision making in aphasia remains to be examined. Future studies will need to determine the unique contribution of brain age relative to other lesion, neuropsychological, and biographical factors associated with language outcomes. As a biomarker of cognitive reserve, brain age is less dependent on factors like language, education, and SES, which frequently influence cognitive testing.[80,81] At the same time, brain age is sensitive both to modifiable environmental factors, such as training[28,39], and changes in cognitive abilities.[82,83] Thus, brain age may be a particularly promising marker of long-term therapy success.

Limitations

The results reported herein, despite being promising, should be interpreted with caution given the novel approach implemented. Several other important limitations of the study design warrant discussion. First, and perhaps most importantly, we included a relatively small sample size that may not support generalization of the results to another sample. Although the sample size is fairly typical for aphasia research[84], the heterogenous nature of language deficits in aphasia reduces statistical power to detect subtle effects of interest.[85] Notwithstanding, it is worth noting that the strength of the association between brain age and language performance in the current study increases our confidence that these findings are not spurious.

Second, estimated brain age was considerably lower on average than chronological age (mean = -3.7 years). This estimate is lower than that reported in most prior studies.[86] There are several potential reasons for this; one potential reason is that cerebrovascular health statistics are generally good in Iceland, especially for women.[87] Importantly, 25/49 participants in the current study were women. Additionally, the Icelandic population has comparatively good access to high quality health care at a low out-of-pocket cost.[88]

Third, one potential criticism of this work is that the enantiomorphic healing process could have introduced artifacts into the brain images that were then used by BrainAgeR to estimate age. We argue that this is unlikely due to our finding that lesion size (and thus the extent to which damaged tissue was replaced with healthy tissue) was not significantly related to estimated brain age differences. Last, the BEST-2 is a coarse measure that may not be sensitive to subtle changes in language function. However, given the substantial functional changes expected in the acute recovery phase[89] in addition to observed improvements across all language tests, this should not affect our results.

Conclusions

In conclusion, our results show for the first time that neuroimaging-based estimation of brain age – as a measure of overall structural integrity of the brain – is strongly associated with language function and recovery following acute stroke. Critically, brain age explained more variability in language performance than chronological age alone. These results hold substantial promise to enhance understanding of the neural bases of aphasia recovery and to improve prognostication in the clinical management of aphasia.

For Review Only

References

1. Engelter ST, Gostynski M, Papa S, et al. Epidemiology of aphasia attributable to first ischemic stroke: incidence, severity, fluency, etiology, and thrombolysis. *Stroke*. 2006;37(6):1379-1384. doi:10.1161/01.STR.0000221815.64093.8c
2. Pedersen PM, Jørgensen HS, Nakayama H, Raaschou HO, Olsen TS. Aphasia in acute stroke: incidence, determinants, and recovery. *Ann Neurol*. 1995;38(4):659-666. doi:10.1002/ana.410380416
3. Watila MM, Balarabe SA. Factors predicting post-stroke aphasia recovery. *J Neurol Sci*. 2015;352(1-2):12-18. doi:10.1016/j.jns.2015.03.020
4. Ali Myzoon, VandenBerg Kathryn, Williams Linda J., et al. Predictors of Poststroke Aphasia Recovery. *Stroke*. 0(0):STROKEAHA.120.031162. doi:10.1161/STROKEAHA.120.031162
5. Lazar RM, Minzer B, Antoniello D, Festa JR, Krakauer JW, Marshall RS. Improvement in aphasia scores after stroke is well predicted by initial severity. *Stroke*. 2010;41(7):1485-1488. doi:10.1161/STROKEAHA.109.577338
6. Benghanem S, Rosso C, Arbizu C, et al. Aphasia outcome: the interactions between initial severity, lesion size and location. *J Neurol*. 2019;266(6):1303-1309. doi:10.1007/s00415-019-09259-3
7. Forkel SJ, Thiebaut de Schotten M, Dell'Acqua F, et al. Anatomical predictors of aphasia recovery: a tractography study of bilateral perisylvian language networks. *Brain*. 2014;137(Pt 7):2027-2039. doi:10.1093/brain/awu113
8. Hillis AE, Beh YY, Sebastian R, et al. Predicting recovery in acute poststroke aphasia. *Annals of Neurology*. 2018;83(3):612-622. doi:10.1002/ana.25184
9. Ellis C, Urban S. Age and aphasia: a review of presence, type, recovery and clinical outcomes. *null*. 2016;23(6):430-439. doi:10.1080/10749357.2016.1150412
10. Toth E, Gersner R, Wilf-Yarkoni A, et al. Age-dependent effects of chronic stress on brain plasticity and depressive behavior. *J Neurochem*. 2008;107(2):522-532. doi:10.1111/j.1471-4159.2008.05642.x
11. Vara H, Muñoz-Cuevas J, Colino A. Age-dependent alterations of long-term synaptic plasticity in thyroid-deficient rats. *Hippocampus*. 2003;13(7):816-825. doi:10.1002/hipo.10132
12. Cole JH, Marioni RE, Harris SE, Deary IJ. Brain age and other bodily "ages": implications for neuropsychiatry. *Mol Psychiatry*. 2019;24(2):266-281. doi:10.1038/s41380-018-0098-1
13. Wrigglesworth J, Ward P, Harding IH, et al. Factors associated with brain ageing - a systematic review. *BMC Neurol*. 2021;21(1):312. doi:10.1186/s12883-021-02331-4
14. Bennett IJ, Madden DJ. Disconnected aging: cerebral white matter integrity and age-related differences in cognition. *Neuroscience*. 2014;276:187-205. doi:10.1016/j.neuroscience.2013.11.026
15. Bonifazi P, Erramuzpe A, Diez I, et al. Structure-function multi-scale connectomics reveals a major role of the fronto-striato-thalamic circuit in brain aging. *Hum Brain Mapp*. 2018;39(12):4663-4677. doi:10.1002/hbm.24312
16. Damoiseaux JS. Effects of aging on functional and structural brain connectivity. *Neuroimage*. 2017;160:32-40. doi:10.1016/j.neuroimage.2017.01.077
17. Fjell AM, Walhovd KB. Structural brain changes in aging: courses, causes and cognitive consequences. *Rev Neurosci*. 2010;21(3):187-221. doi:10.1515/revneuro.2010.21.3.187

For Review Only

18. Fjell AM, Westlye LT, Grydeland H, et al. Accelerating cortical thinning: unique to dementia or universal in aging? *Cereb Cortex*. 2014;24(4):919-934. doi:10.1093/cercor/bhs379
19. Fotenos AF, Snyder AZ, Girton LE, Morris JC, Buckner RL. Normative estimates of cross-sectional and longitudinal brain volume decline in aging and AD. *Neurology*. 2005;64(6):1032-1039. doi:10.1212/01.WNL.0000154530.72969.11
20. Grajauskas LA, Siu W, Medvedev G, Guo H, D'Arcy RCN, Song X. MRI-based evaluation of structural degeneration in the ageing brain: Pathophysiology and assessment. *Ageing Res Rev*. 2019;49:67-82. doi:10.1016/j.arr.2018.11.004
21. Gunning-Dixon FM, Brickman AM, Cheng JC, Alexopoulos GS. Aging of cerebral white matter: a review of MRI findings. *Int J Geriatr Psychiatry*. 2009;24(2):109-117. doi:10.1002/gps.2087
22. Storsve AB, Fjell AM, Tamnes CK, et al. Differential longitudinal changes in cortical thickness, surface area and volume across the adult life span: regions of accelerating and decelerating change. *J Neurosci*. 2014;34(25):8488-8498. doi:10.1523/JNEUROSCI.0391-14.2014
23. Jonsson BA, Bjornsdottir G, Thorgeirsson TE, et al. Brain age prediction using deep learning uncovers associated sequence variants. *Nat Commun*. 2019;10(1):5409. doi:10.1038/s41467-019-13163-9
24. Cole JH, Ritchie SJ, Bastin ME, et al. Brain age predicts mortality. *Mol Psychiatry*. 2018;23(5):1385-1392. doi:10.1038/mp.2017.62
25. Smith SM, Vidaurre D, Alfaro-Almagro F, Nichols TE, Miller KL. Estimation of brain age delta from brain imaging. *Neuroimage*. 2019;200:528-539. doi:10.1016/j.neuroimage.2019.06.017
26. Elliott ML, Belsky DW, Knodt AR, et al. Brain-age in midlife is associated with accelerated biological aging and cognitive decline in a longitudinal birth cohort. *Mol Psychiatry*. 2021;26(8):3829-3838. doi:10.1038/s41380-019-0626-7
27. Cole JH, Leech R, Sharp DJ. Prediction of brain age suggests accelerated atrophy after traumatic brain injury. *Ann Neurol*. 2015;77(4):571-581. doi:10.1002/ana.24367
28. Smith AE, Wade AT, Olds TS, et al. Optimising activity and diet compositions for dementia prevention: Protocol for the ACTIVate prospective longitudinal cohort study [preprint]. 2021. doi:10.1101/2021.07.28.21261299
29. Brett M, Leff AP, Rorden C, Ashburner J. Spatial normalization of brain images with focal lesions using cost function masking. *Neuroimage*. 2001;14(2):486-500. doi:10.1006/nimg.2001.0845
30. Nachev P, Coulthard E, Jäger HR, Kennard C, Husain M. Enantiomorphic normalization of focally lesioned brains. *Neuroimage*. 2008;39(3):1215-1226. doi:10.1016/j.neuroimage.2007.10.002
31. Kristinsson S, Yourganov G, Xiao F, et al. Brain-Derived Neurotrophic Factor Genotype-Specific Differences in Cortical Activation in Chronic Aphasia. *J Speech Lang Hear Res*. 2019;62(11):3923-3936. doi:10.1044/2019_JSLHR-L-RSNP-19-0021
32. Kristinsson S, Zhang W, Rorden C, et al. Machine learning-based multimodal prediction of language outcomes in chronic aphasia. *Hum Brain Mapp*. 2021;42(6):1682-1698. doi:10.1002/hbm.25321

For Review Only

33. Marebwa BK, Fridriksson J, Yourganov G, et al. Chronic post-stroke aphasia severity is determined by fragmentation of residual white matter networks. *Sci Rep.* 2017; 7:8188. doi:10.1038/s41598-017-07607-9
34. Yourganov G, Fridriksson J, Rorden C, Gleichgerrcht E, Bonilha L. Multivariate Connectome-Based Symptom Mapping in Post-Stroke Patients: Networks Supporting Language and Speech. *J Neurosci*. 2016;36(25):6668-6679. doi:10.1523/JNEUROSCI.4396-15.2016
35. Moura LM, Luccas R, de Paiva JPQ, et al. Diffusion Tensor Imaging Biomarkers to Predict Motor Outcomes in Stroke: A Narrative Review. *Front Neurol*. 2019;10:445. doi:10.3389/fneur.2019.00445
36. Ptak R, Bourgeois A, Cavelti S, Doganci N, Schnider A, Iannotti GR. Discrete Patterns of Cross-Hemispheric Functional Connectivity Underlie Impairments of Spatial Cognition after Stroke. *J Neurosci*. 2020;40(34):6638. doi:10.1523/JNEUROSCI.0625-20.2020
37. Salvalaggio A, De Filippo De Grazia M, Zorzi M, Thiebaut de Schotten M, Corbetta M. Post-stroke deficit prediction from lesion and indirect structural and functional disconnection. *Brain*. 2020;143(7):2173-2188. doi:10.1093/brain/awaa156
38. Dubois B, Chupin M, Hampel H, et al. Donepezil decreases annual rate of hippocampal atrophy in suspected prodromal Alzheimer's disease. *Alzheimers Dement*. 2015;11(9):1041-1049. doi:10.1016/j.jalz.2014.10.003
39. Johnson L, Werden E, Shirbin C, et al. The Post Ischaemic Stroke Cardiovascular Exercise Study: Protocol for a randomised controlled trial of fitness training for brain health. *Eur Stroke J*. 2018;3(4):379-386. doi:10.1177/2396987318785845
40. Langeskov-Christensen M, Grøndahl Hvid L, Nygaard MKE, et al. Efficacy of High-Intensity Aerobic Exercise on Brain MRI Measures in Multiple Sclerosis. *Neurology*. 2021;96(2):e203-e213. doi:10.1212/WNL.0000000000011241
41. Egorova N, Liem F, Hachinski V, Brodtmann A. Predicted Brain Age After Stroke. *Front Aging Neurosci*. 2019;11:348-348. doi:10.3389/fnagi.2019.00348
42. Richard G, Kolskår K, Ulrichsen KM, et al. Brain age prediction in stroke patients: Highly reliable but limited sensitivity to cognitive performance and response to cognitive training. *Neuroimage Clin*. 2020;25:102159-102159. doi:10.1016/j.nicl.2019.102159
43. de Lange AMG, Anatürk M, Suri S, et al. Multimodal brain-age prediction and cardiovascular risk: The Whitehall II MRI sub-study. *NeuroImage*. 2020;222:117292. doi:10.1016/j.neuroimage.2020.117292
44. Franke K, Gaser C. Ten Years of BrainAGE as a Neuroimaging Biomarker of Brain Aging: What Insights Have We Gained? *Front Neurol*. 2019;10:789. doi:10.3389/fneur.2019.00789
45. Bretzner M, Bonkhoff A, Schirmer M, et al. Radiomics Derived Brain Age Predicts Functional Outcome After Acute Ischemic Stroke [preprint]. 2021. doi:10.21203/rs.3.rs-923769/v1
46. Brodtmann A, Werden E, Khlif MS, et al. Neurodegeneration Over 3 Years Following Ischaemic Stroke: Findings From the Cognition and Neocortical Volume After Stroke Study. *Front Neurol*. 2021;12:754204. doi:10.3389/fneur.2021.754204

For Review Only

47. Veldsman M, Cheng HJ, Ji F, et al. Degeneration of structural brain networks is associated with cognitive decline after ischaemic stroke. *Brain Communications*. 2020;2(2):fcaa155. doi:10.1093/braincomms/fcaa155
48. Veldsman M, Werden E, Egorova N, Khlif MS, Brodtmann A. Microstructural degeneration and cerebrovascular risk burden underlying executive dysfunction after stroke. *Scientific Reports*. 2020;10(1):17911. doi:10.1038/s41598-020-75074-w
49. Magnusdottir S, Fillmore P, den Ouden DB, et al. Damage to left anterior temporal cortex predicts impairment of complex syntactic processing: a lesion-symptom mapping study. *Hum Brain Mapp*. 2013;34(10):2715-2723. doi:10.1002/hbm.22096
50. Kristinsson S, Thors H, Yourganov G, et al. Brain Damage Associated with Impaired Sentence Processing in Acute Aphasia. *J Cogn Neurosci*. 2020;32(2):256-271. doi:10.1162/jocn_a_01478
51. Fitch-West J, Sands ES, Ross-Swain D. Bedside Evaluation Screening Test–Second Edition (BEST-2). Austin, TX: Pro-Ed. 1998.
52. Kertesz A. *Western aphasia battery–Revised (WAB-R)*. San Antonio, TX: Pearson. 2007.
53. Wallace SJ, Worrall L, Rose T, et al. A core outcome set for aphasia treatment research: The ROMA consensus statement. *Int J Stroke*. 2019;14(2):180-185. doi:10.1177/1747493018806200
54. Fridriksson J, Holland AL, Coull BM, Plante E, Trouard TP, Beeson P. Aphasia severity: Association with cerebral perfusion and diffusion. *Aphasiology*. 2002;16(9):859-871. doi:10.1080/02687030244000347
55. Li X, Morgan PS, Ashburner J, Smith J, Rorden C. The first step for neuroimaging data analysis: DICOM to NIfTI conversion. *J Neurosci Methods*. 2016;264:47-56. doi:10.1016/j.jneumeth.2016.03.001
56. Rorden C, Brett M. Stereotaxic display of brain lesions. *Behav Neurol*. 2000;12(4):191-200. doi:10.1155/2000/421719
57. Rorden C, Bonilha L, Fridriksson J, Bender B, Karnath HO. Age-specific CT and MRI templates for spatial normalization. *Neuroimage*. 2012;61(4):957-965. doi:10.1016/j.neuroimage.2012.03.020
58. Ashburner J, Friston KJ. Unified segmentation. *NeuroImage*. 2005;26(3):839-851. doi:10.1016/j.neuroimage.2005.02.018
59. IBM Corp. IBM SPSS Statistics for Windows, Version 28.0. Armonk, NY: IBM Corp. 2021.
60. Sibille E. Molecular aging of the brain, neuroplasticity, and vulnerability to depression and other brain-related disorders. *Dialogues Clin Neurosci*. 2013;15(1):53-65. doi:10.31887/DCNS.2013.15.1/esibille
61. Del Maschio N, Sulpizio S, Gallo F, Fedeli D, Weekes BS, Abutalebi J. Neuroplasticity across the lifespan and aging effects in bilinguals and monolinguals. *Brain Cogn*. 2018;125:118-126. doi:10.1016/j.bandc.2018.06.007
62. Bishop NA, Lu T, Yankner BA. Neural mechanisms of ageing and cognitive decline. *Nature*. 2010;464(7288):529-535. doi:10.1038/nature08983
63. Deary IJ, Corley J, Gow AJ, et al. Age-associated cognitive decline. *Br Med Bull*. 2009;92:135-152. doi:10.1093/bmb/ldp033

For Peer Review Only

64. Shafto MA, Tyler LK. Language in the aging brain: the network dynamics of cognitive decline and preservation. *Science*. 2014;346(6209):583-587. doi:10.1126/science.1254404
65. Etherton MR, Wu O, Rost NS. Recent Advances in Leukoaraiosis: White Matter Structural Integrity and Functional Outcomes after Acute Ischemic Stroke. *Curr Cardiol Rep*. 2016;18(12):123. doi:10.1007/s11886-016-0803-0
66. van Meer MPA, Otte WM, van der Marel K, et al. Extent of bilateral neuronal network reorganization and functional recovery in relation to stroke severity. *J Neurosci*. 2012;32(13):4495-4507. doi:10.1523/JNEUROSCI.3662-11.2012
67. Good CD, Johnsrude IS, Ashburner J, Henson RN, Friston KJ, Frackowiak RS. A voxel-based morphometric study of ageing in 465 normal adult human brains. *Neuroimage*. 2001;14(1 Pt 1):21-36. doi:10.1006/nimg.2001.0786
68. Fridriksson J, Hillis AE. Current Approaches to the Treatment of Post-Stroke Aphasia. *J Stroke*. 2021;23(2):183-201. doi:10.5853/jos.2020.05015
69. Kirkwood TBL. Understanding the odd science of aging. *Cell*. 2005;120(4):437-447. doi:10.1016/j.cell.2005.01.027
70. Kirkwood TBL. A systematic look at an old problem. *Nature*. 2008;451(7179):644-647. doi:10.1038/451644a
71. Cherbuin N, Walsh EI, Shaw M, et al. Optimal Blood Pressure Keeps Our Brains Younger. *Front Aging Neurosci*. 2021;13:694982-694982. doi:10.3389/fnagi.2021.694982
72. Cox S, Lyall D, Ritchie S, et al. Associations between vascular risk factors and brain MRI indices in UK Biobank. *European Heart Journal*. Published online February 13, 2019. doi:10.1093/eurheartj/ehz100
73. Launer LJ, Lewis CE, Schreiner PJ, et al. Vascular factors and multiple measures of early brain health: CARDIA brain MRI study. *PLoS One*. 2015;10(3):e0122138. doi:10.1371/journal.pone.0122138
74. Gaser C, Franke K, Klöppel S, Koutsouleris N, Sauer H. BrainAGE in Mild Cognitive Impaired Patients: Predicting the Conversion to Alzheimer's Disease. *PLoS One*. 2013;8(6):e67346. doi:10.1371/journal.pone.0067346
75. Pardoe HR, Cole JH, Blackmon K, Thesen T, Kuzniecky R. Structural brain changes in medically refractory focal epilepsy resemble premature brain aging. *Epilepsy Res*. 2017;133:28-32. doi:10.1016/j.eplepsyres.2017.03.007
76. Schnack HG, van Haren NEM, Nieuwenhuis M, Hulshoff Pol HE, Cahn W, Kahn RS. Accelerated Brain Aging in Schizophrenia: A Longitudinal Pattern Recognition Study. *Am J Psychiatry*. 2016;173(6):607-616. doi:10.1176/appi.ajp.2015.15070922
77. Schlaug G, Marchina S, Norton A. Evidence for plasticity in white-matter tracts of patients with chronic Broca's aphasia undergoing intense intonation-based speech therapy. *Ann N Y Acad Sci*. 2009;1169:385-394. doi:10.1111/j.1749-6632.2009.04587.x
78. Bonilha L, Gleichgerrcht E, Nesland T, Rorden C, Fridriksson J. Success of Anomia Treatment in Aphasia Is Associated With Preserved Architecture of Global and Left Temporal Lobe Structural Networks. *Neurorehabil Neural Repair*. 2016;30(3):266-279. doi:10.1177/1545968315593808
79. Cheng BBY, Worrall LE, Copland DA, Wallace SJ. Prognostication in post-stroke aphasia: How do speech pathologists formulate and deliver information about recovery? *Int J Lang Commun Disord*. 2020;55(4):520-536. doi:10.1111/1460-6984.12534

For Review Only

80. Makin SD, Doubal FN, Shuler K, et al. The impact of early-life intelligence quotient on post stroke cognitive impairment. *Eur Stroke J*. 2018;3(2):145-156. doi:10.1177/2396987317750517
81. Harrison SL, Sajjad A, Bramer WM, Ikram MA, Tiemeier H, Stephan BCM. Exploring strategies to operationalize cognitive reserve: A systematic review of reviews. *J Clin Exp Neuropsychol*. 2015;37(3):253-264. doi:10.1080/13803395.2014.1002759
82. Jack CRJ, Lowe VJ, Weigand SD, et al. Serial PIB and MRI in normal, mild cognitive impairment and Alzheimer's disease: implications for sequence of pathological events in Alzheimer's disease. *Brain*. 2009;132(Pt 5):1355-1365. doi:10.1093/brain/awp062
83. Jack CRJ, Knopman DS, Jagust WJ, et al. Hypothetical model of dynamic biomarkers of the Alzheimer's pathological cascade. *Lancet Neurol*. 2010;9(1):119-128. doi:10.1016/S1474-4422(09)70299-6
84. Wilson SM, Schneck SM. Neuroplasticity in post-stroke aphasia: A systematic review and meta-analysis of functional imaging studies of reorganization of language processing. *Neurobiol Lang (Camb)*. 2021;2(1):22-82. doi:10.1162/nol_a_00025
85. Lorca-Puls DL, Gajardo-Vidal A, White J, et al. The impact of sample size on the reproducibility of voxel-based lesion-deficit mappings. *Neuropsychologia*. 2018;115:101-111. doi:10.1016/j.neuropsychologia.2018.03.014
86. Baecker L, Garcia-Dias R, Vieira S, Scarpazza C, Mechelli A. Machine learning for brain age prediction: Introduction to methods and clinical applications. *EBioMedicine*. 2021;72:103600. doi:10.1016/j.ebiom.2021.103600
87. Levi F, Lucchini F, Negri E, La Vecchia C. Trends in mortality from cardiovascular and cerebrovascular diseases in Europe and other areas of the world. *Heart*. 2002;88(2):119-124. doi:10.1136/heart.88.2.119
88. Gustafsdottir SS, Fenger K, Halldorsdottir S, Bjarnason T. Social justice, access and quality of healthcare in an age of austerity: users' perspective from rural Iceland. *Int J Circumpolar Health*. 2017;76(1):1347476. doi:10.1080/22423982.2017.1347476
89. Laska AC, Hellblom A, Murray V, Kahan T, Von Arbin M. Aphasia in acute stroke and relation to outcome. *J Intern Med*. 2001;249(5):413-422. doi:10.1046/j.1365-2796.2001.00812.x

For Review Only

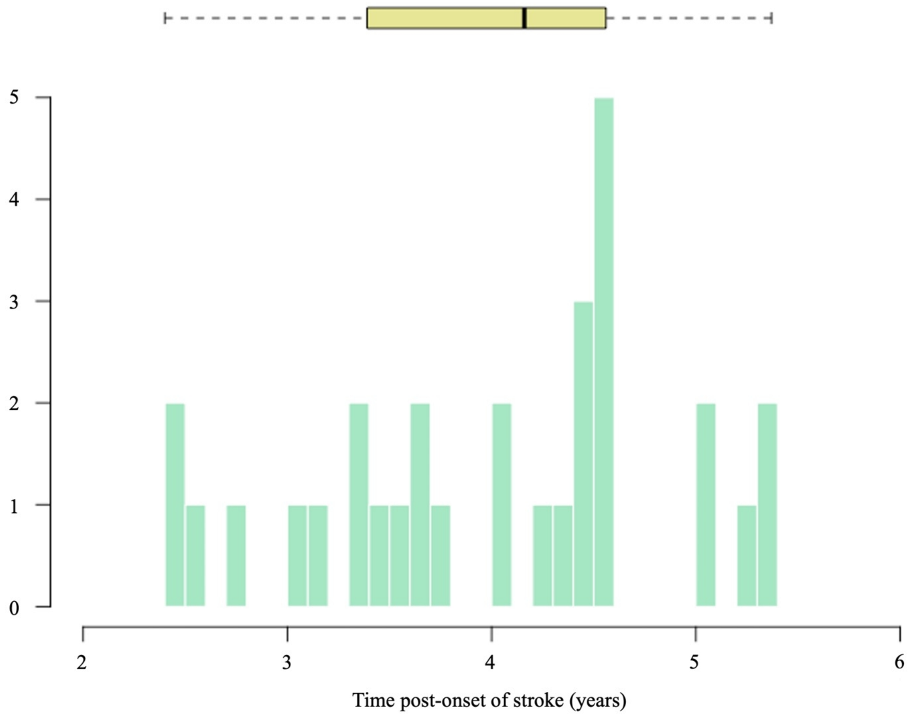

Figure 1. Time post-onset of left hemisphere stroke in years at retesting (min = 2.4y, max = 5.4y).

115x93mm (330 x 330 DPI)

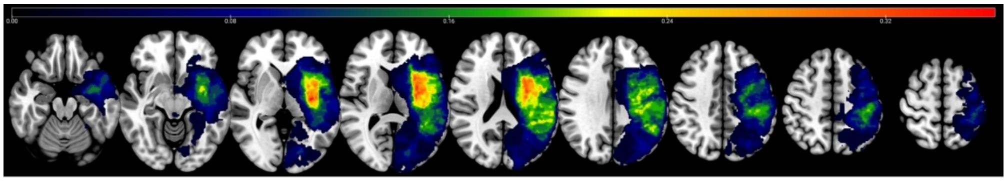

Figure 2. Lesion overlap across participants. The colorbar represents proportional overlap (max = 37% overlap).

165x28mm (330 x 330 DPI)

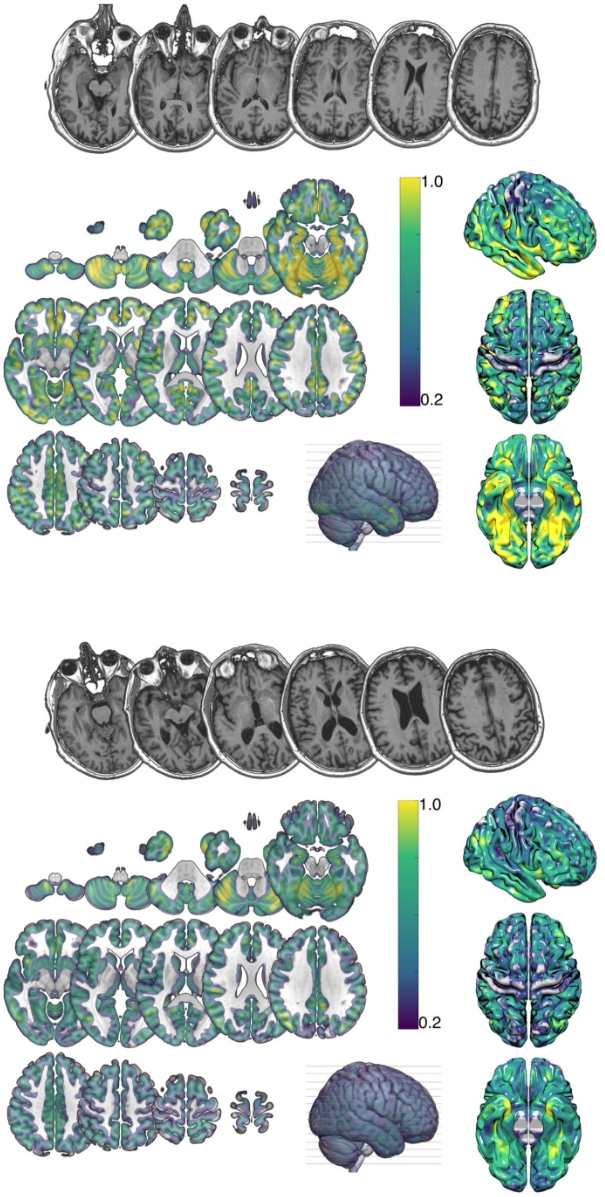

Figure 3. Probabilistic gray matter estimates from two representative participants. Top panel: Male, chronological age = 60.2 years, brain age = 36.9 years, PBAG = -.39); bottom panel: Male, chronological age = 62.4 years, brain age = 71.7, PBAG = .15). The colorbar represents the probabilistic measure of gray matter volume (darker colors suggesting less gray matter).

108x208mm (330 x 330 DPI)

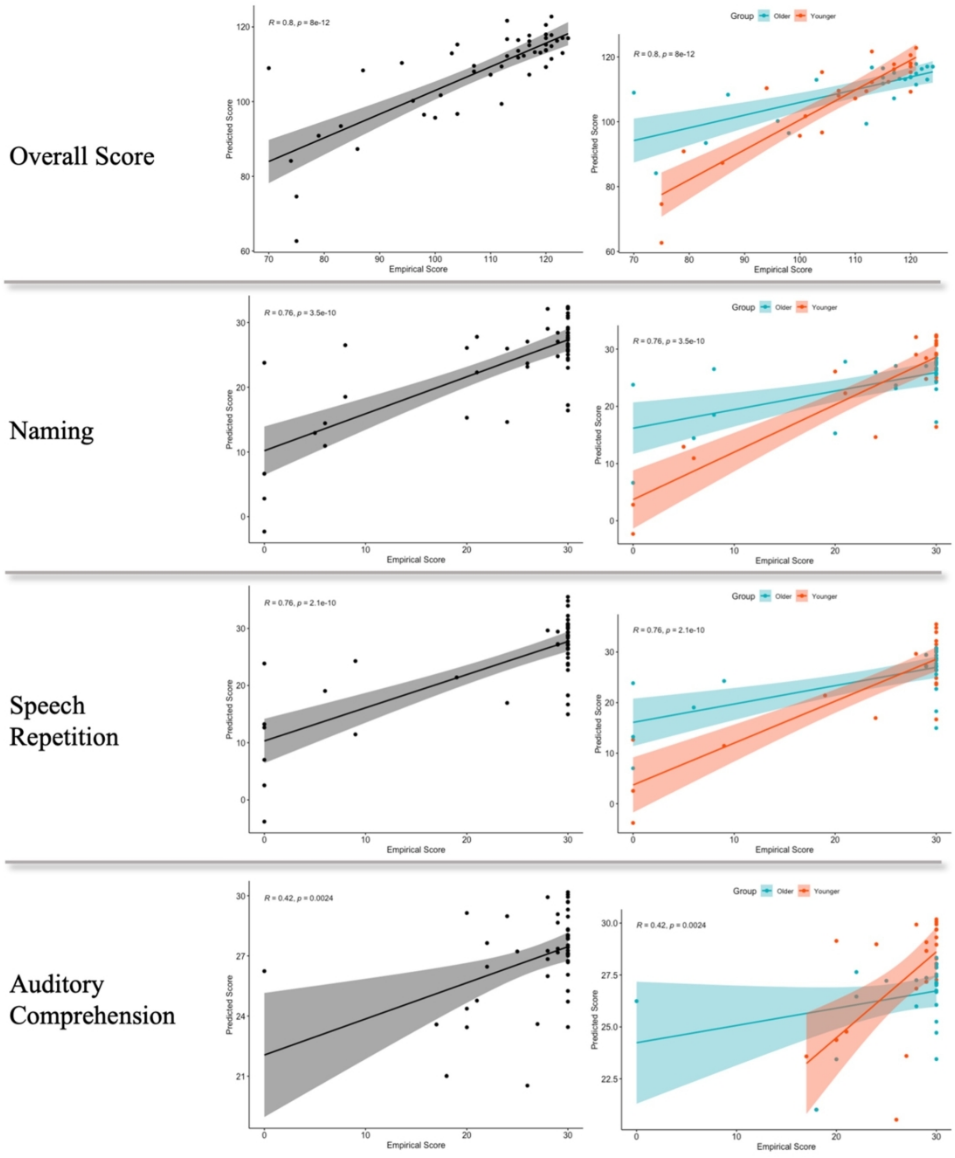

Figure 4. Actual vs. predicted language scores at stroke onset. Multiple regression models included lesion volume, chronological age, and brain age as independent terms. Left panel: full sample; right panel: color-coded based on older/younger estimated brain age.

166x197mm (330 x 330 DPI)

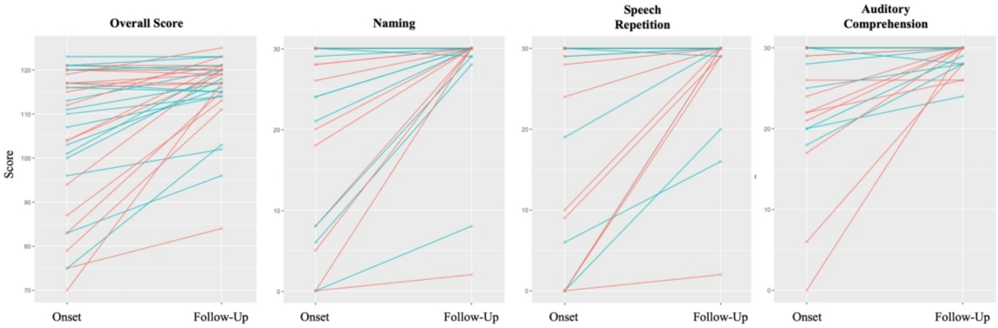

Figure 5. Longitudinal recovery across language domains. Line colors represent brain age; blue = older, orange = younger.

165x62mm (330 x 330 DPI)
