# Supplementary material for: Brain age predicts long-term recovery in post-stroke aphasia
Source: Brain Commun. 2022 Oct 6;4(5):fcac252. doi: 10.1093/braincomms/fcac252 (PMC9576153; doi:10.1093/braincomms/fcac252)
Supplement: fcac252_Supplementary_Data [file fcac252_supplementary_data.zip › Supplementary_material.pdf]

Supplementary Tables

	F	t	ß	η^2	*p*
			**Overall Score**		
Model	2.22			$R^2 = .20$	.110
Lesion volume	6.63	2.58	.50	.20	.016
Chronological age	1.20	1.09	.21	.04	.284
PBAD	.43	.66	.12	.02	.518
			**Naming**		
Model	2.02			$R^2 = .19$	.136
Lesion volume	3.22	1.79	.35	.11	.084
Chronological age	1.81	1.35	.26	.07	.190
PBAD	3.51	1.87	.34	.12	.072
			**Speech Repetition**		
Model	4.23			$R^2 = .33$	.015
Lesion volume	9.14	3.02	.54	.26	.006
Chronological age	.92	.96	.17	.03	.346
PBAD	6.49	2.55	.42	.20	.017*
			**Auditory Comprehension**		
Model	1.36			$R^2 = .14$	.278
Lesion volume	2.74	1.66	.34	.10	.110
Chronological age	2.13	1.46	.29	.08	.156
PBAD	1.12	1.06	.20	.04	.299

Supplementary Table 1. General Linear Models Predicting Raw Change in Language Performance from Stroke Onset to Follow-Up Without Adjustment for Baseline Severity (BEST). $^*p < .05$.

	F	t	ß	η^2	*p*
			**Overall Score**		
Model	13.53			.61 (R^2 = .57)	<.001**
Lesion volume	34.89	-5.91	-.83	.57	<.001**
Chronological age	.28	-.53	-.07	.01	.599
PBAD	4.98	-2.23	-.28	.16	.034*
			**Naming**		
Model	8.51			.57 (R^2 = .51)	<.001**
Lesion volume	23.76	-4.87	-.85	.56	<.001**
Chronological age	2.12	-1.46	-.25	.10	.162
PBAD	4.14	-2.04	-.31	.18	.056
			**Speech Repetition**		
Model	8.38			.57 (R^2 = .50)	<.001**
Lesion volume	19.80	-4.45	-.78	.51	<.001**
Chronological age	.28	-.53	-.09	.01	.605
PBAD	6.32	-2.51	-.39	.25	.021*
			**Auditory Comprehension**		
Model	1.03			.14 (R^2 = .00)	.140
Lesion volume	2.03	-1.43	-.35	.10	.170
Chronological age	.50	-.71	-.17	.03	.489
PBAD	1.73	-1.32	-.29	.08	.204

Supplementary Table 2. General Linear Models predicting language performance at stroke onset after removing ceiling scores. *$p < .05$, **$p < .01$.

	F	t	ß	η^2	*p*
			**Overall Score**		
Model	37.63			.85 (R^2 = .83)	<.001**
Baseline score	110.88	-10.53	-1.17	.80	<.001**
Lesion volume	11.86	-3.44	-.42	.31	.002**
Chronological age	2.04	1.43	.12	.07	.165
PBAD	5.91	-2.43	-.20	.18	.022*
			**Naming**		
Model	13.16			.83 (R^2 = .76)	<.001
Baseline score	38.24	-6.18	-1.01	.78	<.001
Lesion volume	10.48	-3.24	-.66	.49	.008
Chronological age	.43	.66	.10	.04	.523
PBAD	.04	-.19	-.03	.00	.850
			**Speech Repetition**		
Model	4.95			.71 (R^2 = .57)	.026
Baseline score	7.62	-2.76	-.88	.49	.025
Lesion volume	.30	-.55	-.23	.04	.598
Chronological age	.66	.81	.20	.08	.440
PBAD	.03	.18	.06	.00	.863
			**Auditory Comprehension**		
Model	69.84			.97 (R^2 = .95)	<.001
Baseline score	181.56	-13.47	-1.05	.95	<.001
Lesion volume	4.56	-2.14	-.18	.31	.058
Chronological age	1.17	-1.08	-.10	.11	.305
PBAD	3.09	-1.76	-.13	.24	.109

Supplementary Table 3. General Linear Models predicting raw change in language performance from stroke onset to follow-up after removing baseline ceiling scores. *$p < .05$, **$p < .01$.
