# Supplementary material for: Brain age predicts long-term recovery in post-stroke aphasia
Source: Brain Commun. 2022 Oct 6;4(5):fcac252. doi: 10.1093/braincomms/fcac252 (PMC9576153; doi:10.1093/braincomms/fcac252)
Supplement: fcac252_Supplementary_Data [file Original Submission - Author's Response.pdf › Original Submission - Author's Response.pdf]

Center for the study of aphasia recovery (C-STAR)
Arnold School of Public Health
University of South Carolina

Columbia, SC

07/22/2022

Prof. Alfred K. Njamnshi, Ph.D.
Associate Editor of Brain Communications

Dear Dr. Njamnshi,

We thank you and the reviewers for the detailed review of our manuscript "Brain Age Predicts Long-Term Recovery in Post-Stroke Aphasia" (BRAINCOM-2022-082). We appreciate the reviewers' positive comments and helpful suggestions to improve our manuscript.

In response to the editor's and reviewers' comments, we have made substantial changes to the manuscript and below we provide point-by-point responses to all comments and suggestions. These include revisions on our analysis pipeline and clarifications regarding several critical aspects of our methodology. More specifically, we made one substantial revision in response to the second reviewer's major concern. We modified the analysis pipeline and reran all analyses pertaining to the longitudinal data. As opposed to the longitudinal models in the original manuscript, we used raw change scores between language measures obtained at stroke onset and at follow-up as dependent variables in multiple regression models adjusted for baseline severity. We are happy to report that this more robust approach nonetheless suggests that estimated brain age is strongly associated with long-term recovery of language function. In addition, we implemented a multitude of minor revisions suggested by the second reviewer.

All revisions are highlighted in the revised manuscript document and supplementary documents. Overall, we believe the implementation of recommended changes represent substantial improvements of our manuscript. We hope you concur.

On behalf of the authors,
Sigfus Kristinsson

Reviewer comments and responses

Reviewer 1

Comments to the Author
Kristinsson and colleagues present a new method to evaluate a usual suspect in the recovery of post-stroke aphasia, which remains elusive: brain age. The evaluation of this method in a small sample shows its feasibility and opens the door for it to be evaluated further. The studies has its limitations, which the authors acknowledge, but these should not preclude the publication of the study.

> **Authors' Response:** We thank the reviewer for the positive feedback on our manuscript. Conceptually, examining the causal relationship between estimated brain age and language recovery after stroke is a novelty. However, the relationship between brain age and various other cognitive functions, as well as neurologic diseases, has been a subject of numerous prior studies (e.g., as cited in our manuscript: Elliott. et al., 2021; Fjell. &. Walhvd, 2010; Wrigglesworth et al., 2021). Generally, most prior findings are consistent with the notion that estimated brain age serves as a biomarker for the structural and functional integrity of the adult brain and, by extension, is related to cognition and early onset of neurodegenerative disorders.
>
> Despite these promising results, our study certainly has its limitations (e.g., how to treat the lesioned areas), which we tried to acknowledge in the manuscript. This is not uncommon when a novel approach is implemented within an established field of study. We believe that future studies will investigate the predictive value for various language outcomes (we are aware of at least two other such studies to be submitted soon), and we encourage authors to keep these limitations in mind.

Reviewer 2

Comments to the Author
In the manuscript, Kristinsson et al. aimed to use brain age predicting stroke patients' language function and long-term recovery. They collected 49 participants with left stroke. Brain age was estimated through machine learning algorithm. The multiple regression results revealed that brain age is an effective biomarker for stroke's language function and recovery, irrespective of the influence of chronological age and lesion volume. The study has a relatively large sample size for follow-up analysis. The method is solid, the result is clear, the manuscript is well-written. Most importantly, the finding may make progress for future clinical application, as stroke prognosis is still hard to predict. Following are several concerns about it.

> **Authors' Response:** We thank the reviewer for the positive overall feedback. Our study is a proof-of-principle study within the field of aphasiology. For this reason, we were intentional about implementing a simple, albeit robust, study design and statistical approaches. We believe that in the revised manuscript, we have achieved this goal.
>
> More broadly, we recognize that prognosis remains a substantial problem in aphasia care. Anecdotally, we hear from our study participants that the message they got when they left the hospital was: "You'll recover some of your pre-stroke language function in the next few months, but little changes are expected after the first 6-12 months." This message shows how short we've come in terms of prognostication. Identifying robust biomarkers will be critical to improve clinical prognostication. The primary strength of our study in this context is that brain age was estimated from clinical scans, which shows promise for future clinical application.

Point-by-Point Responses

1. The main concern is the recovery analysis. Why did the authors look at the main effects of the predictors? If want to control for baseline severity, may need to look at the interaction between predictors and behavioral performance.

> **Authors' Response:** We thank the reviewer for raising a valid concern. In the original version of our manuscript, we examined the main effects of lesion volume, chronological age, and proportional brain age difference (PBAD) on language outcomes assessed at stroke onset and follow-up with a repeated-measures analysis of variance. While we observed a significant main effect of PBAD in 2/4 models (overall score, speech repetition), the analysis did not consider the effect of time. Nonetheless, the manuscript concluded that PBAD was significantly associated with long-term language recovery. A more appropriate interpretation of these results would be that PBAD was significantly associated with language function at onset and follow-up, while accounting for the within-subject correlation between the two timepoints.
>
> In the revised manuscript, we re-designed the analysis pipeline for the recovery models to better address the second study aim. Instead of the repeated-measures design, we constructed multiple linear regression models to predict raw change in language performance from onset to follow-up (DV = $BEST_{Follow\text{-}Up} – BEST_{Onset}$). Each recovery model included the same terms as the stroke onset models constructed to address our first aim (lesion volume, chronological age, and PBAD), in addition to subtest-specific baseline BEST scores (i.e., BEST Naming Score at onset was included in the model predicting raw change in naming performance). Changes have been made to the Methods, Results, and Discussion where appropriate. All changes are highlighted yellow. We believe this approach remediates the reviewer's concern and strengthens our manuscript considerably.
>
> In addition, we incorporated a *post hoc* analysis replicating the same recovery models without adjustment for baseline performance to enable direct comparison to the cross-sectional stroke onset models. As a secondary analysis, these results are noted in the Results section, and the full models are shown in Supplementary Table 1 in the revised manuscript.

2. Whether the model including brain age is significantly improved compared with model with lesion volume only?

> **Authors' Response:** The reviewer brings up an important question. We aimed to compare the predictive value of chronological age and PBAD on language performance at stroke onset and recovery of language function. However, we recognize that the

answer to the research question holds limited clinical relevance if neither measure explains variability in language measures not captured by lesion volume alone. To address this question, we performed a statistical comparison between models based on lesion volume alone and models based on lesion volume and PBAD. The results are shown in the table below:

Model	Overall	Naming	Repetition	Comprehension
Onset Models	Resid. Deviance	Resid. Deviance	Resid. Deviance	Resid. Deviance
~ Lesion	4,651.7	2,525.8	2,816.9	1,326.5
~ Lesion+PBAD	4,150.8	2,310.8	2,395.3	1,276.3
	$p > X^2 =$ .018	$p > X^2 =$ .039	$p > X^2 =$ .004	$p > X^2 =$ .178
Recovery Mod.[1]	Resid. Deviance	Resid. Deviance	Resid. Deviance	Resid. Deviance
~ Lesion	927.4	350.9	521.9	49.4
~ Lesion+PBAD	756.4	350.9	521.8	44.9
	$p > X^2 =$ .015	$p > X^2 =$ .956	$p > X^2 =$ .954	$p > X^2 =$ .105

Model Comparison*. For each language measure, the residual deviance of a model based on lesion volume alone was compared to the residual deviance of a model based on lesion volume and PBAD. Significant differences are highlighted yellow. [1]Adjusted for baseline BEST scores.*

As shown in the table, models based on lesion volume and PBAD yield significantly lower residual deviance than the models based on lesion volume alone in 3/4 cases where the dependent variable was language performance at stroke onset. In the recovery models, the residual deviance was significantly reduced only for the prediction of change in Overall Score, although the difference was marginally significant for Auditory Comprehension.

Importantly, we ran the same model comparisons to test whether including chronological age would significantly improve model predictions based on lesion volume alone. These analyses revealed that chronological age did not significantly improve any of the 8 models based on lesion volume (all $p > .05$). Therefore, our results strongly suggest that the proportional difference between estimated brain age and chronological age accounts for unique variability in language performance and language recovery after a single-event left hemisphere stroke.

3. According to figure 4, many points are on the ceiling or floor for individual language tasks, making the scatter patterns odd. Do they heavily influence the result?

Authors' Response: There are only a handful of individuals whose performance is at floor level, but a large portion of participants scored at ceiling at stroke onset. The reason is simply that participants were not required to have a diagnosis of aphasia to be included in the study. Participants only had to have occurred a single-event stroke to the left

hemisphere (and no history of prior strokes) and to seek medical attention at the local hospital for their symptoms. Thus, even though the distribution of language scores may present an inconvenience in a research context, this distribution represents routine clinical reality.

In an effort to adequately address the reviewer's question, we re-ran all models excluding participants with baseline scores at ceiling, both in predicting language scores at stroke onset and language recovery. Briefly, removing these individuals had minimal effects on our results; the models were somewhat less robust due to substantial cut in the number of participants, but the same main effects emerged consistently. These analyses have been added to the revised manuscript (referenced in the Discussion section). The full models are presented in Supplementary Tables 2 and 3.

4. What the information can tell by splitting participants in figure 4? The authors may want to indicate younger group had better performance? But it doesn't look like that.

Authors' Response: We thank the reviewer for bringing up this issue. In the original version of the manuscript, Figure 4 showed empirical vs. predicted language scores for two groups of participants based on their estimated brain age (relatively younger/older brain age). Our intention was to show that the distribution of predicted outcomes was uniform across the study sample, as opposed to being biased based on whether estimated brain age was higher or lower than chronological age. However, we agree with the reviewer that the figure does not present the intended information in a clear manner. Therefore, we discarded the group division and revised the figure to only show empirical vs. predicted language outcomes, along with Pearson's correlation, in the full sample. We believe the updated figure clearly demonstrates that there is no obvious bias in the prediction models and, thus, that this revision constitutes a meaningful improvement of our manuscript.

5. In discussion, the authors can discuss different results of individual language measures and give some explanations.

Authors' Response: We acknowledge the importance of the reviewer's observation. In the original manuscript, we were intentional about not interpreting the individual language measure in detail since the BEST is a short and fairly coarse measure of language. Moreover, we are very mindful that this is the first study within the field of aphasiology to apply the brain age measure and, as such, we remain skeptical about drawing conclusions about specific subtests. We believe that these findings are highly important and promising in terms of enhancing prognostication in the clinical management of aphasia, but as our manuscript describes a proof-of-principle study, we have emphasized that brain age may be a future biomarker for language recovery broadly speaking, although future research will be left to demonstrate its sensitivity to predict

changes in specific language functions. Furthermore, we highlighted another obvious, albeit important, element in our study – i.e., that we were able to estimate brain age using clinical-grade brain scans, which has remained an unrealistic goal in clinical practice thus far. Notwithstanding, based on the reviewer's comment, we elaborated on the tasks for which brain age (PBAD) emerged as a significant predictor of in the Discussion section. We hope this change meets the reviewer's expectation; otherwise, we are certainly happy to discuss the individual language measures in greater details.

6. P7: Why did brain/chronological age negatively correlate with lesion volume? Referring to Q9, shouldn't they be positive or no significant relation? Is this a general result pattern or specific for this cohort only?

Authors' Response: We appreciate the reviewer's insightful questions. Unfortunately, our data does not enable us to definitively determine why age was negatively correlated with lesion volume in this particular sample. Multiple prior studies have observed a multidimensional relationship between age and lesion volume, where interactions with variable cardiovascular risk factors (e.g., diabetes, stress, smoking) and other health factors (e.g., mobility) can have mediating effects.[1,2,3] We recruited a nonselective sample at a local hospital and participants' ages ranged from early 30s and 40s to late 70s and 80s. As shown in the figure below, our data indicates somewhat of a linear relationship, but the relationship is not unaffected by a handful of datapoints on each tail end of the age range. One possible explanation is that the younger individuals in our study had more cardiovascular risk factors present, which put them at risk of incurring stroke earlier on in life and, potentially, at greater risk for severe strokes, whereas the oldest individuals may have been relatively healthy prior to their strokes. This seems like a reasonable possibility given that participants were recruited only if they incurred their first-ever stroke. Indeed, if this is the case, we would expect the difference between estimated brain age and chronological age to be a stronger predictor of language outcomes than chronological age since the former measure is related to factors that affect brain health.

---

[1] Lövblad, K. O., Baird, A. E., Schlaug, G., Benfield, A., Siewert, B., Voetsch, B., Connor, A., Burzynski, C., Edelman, R. R., & Warach, S. (1997). Ischemic lesion volumes in acute stroke by diffusion-weighted magnetic resonance imaging correlate with clinical outcome. *Annals of neurology*, *42*(2), 164–170. https://doi.org/10.1002/ana.410420206

[2] Umarova, R. M., Sperber, C., Kaller, C. P., Schmidt, C., Urbach, H., Klöppel, S., Weiller, C., & Karnath, H. O. (2019). Cognitive reserve impacts on disability and cognitive deficits in acute stroke. *Journal of neurology*, *266*(10), 2495–2504. https://doi.org/10.1007/s00415-019-09442-6

[3] Umarova, R.M., Schumacher, L.V., Schmidt, C.S.M. *et al.* Interaction between cognitive reserve and age moderates effect of lesion load on stroke outcome. *Sci Rep* **11,** 4478 (2021). https://doi.org/10.1038/s41598-021-83927-1

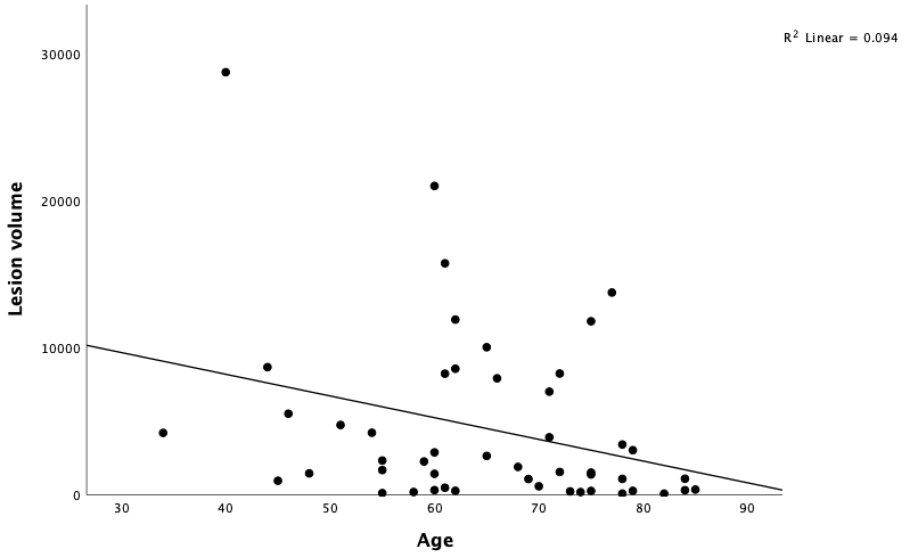

Scatterplot of Age vs. Lesion Volume*. At stroke onset, we observed a weak negative correlation between age and lesion volume.*

7. P5: All the participants were first-time stroke? Do they have any chronic lesions?

Authors' Response: All participants in the study were recruited after incurring a single stroke, with no history of prior strokes and no chronic lesions present on CT/MRI scans. We have clarified this in the revised manuscript by changing the fourth inclusion criteria to read: "(4) had no history of prior stroke, major psychiatric illness or other neurologic impairment affecting the brain."

8. P5: may need to report the TPO of the acute cohort. In addition, why is acute lesion demarcated on FLAIR not DWI?

Authors' Response: All participants were scanned upon hospital admission or as soon after admission as possible, within a period of three days. Scans were acquired for clinical purposes to determine prognosis and to guide treatment planning.

Lesions were demarcated on FLAIR images as these images had higher resolution than the DWI images. Since FLAIR is optimized to detect white matter hyperintensities, which do not show well on diffusion scans due to the lower resolution, lesion maps were compared to DWI images to ensure lesioned areas appeared hyperintense on both types of images. If an area appeared as hyperintense on FLAIR, but not on diffusion scans, then we assumed it was not the lesion. Therefore, all lesion maps are in the same resolution

and space as the FLAIR images, but diffusion images were used to guide manual lesion drawing. We have clarified this in the Methods section of the manuscript.

9. P3, line 15-16; P10, line 29-30: "older individuals are more likely to present with severe aphasia, which may negate the direct association between age and recovery." Is it the same situation for brain age, given brain and chronological ages are highly related? How did the study overcome this issue? Smaller age variability or using the proportional difference?

Authors' Response: We thank the reviewer for these important questions. First, we wish to note that we modified the wording of the sentence quoted by the reviewer as we believe the word "negate" can be misleading in this context. In the revised manuscript, the sentence has been modified as follows: "However, older individuals are more likely to present with severe aphasia, which may indicate that the effect of age on recovery is confounded by aphasia severity, at least in some age groups."

Unfortunately, there is no existing literature on the association between estimated brain age and aphasia severity. In fact, the first study using brain age to examine language performance in stroke remains to be published altogether. Notwithstanding, given the strong correlation between chronological age and brain age, it is reasonable to expect a similar relationship between brain age and aphasia severity. However, brain age is estimated based on cortical atrophy and, as such, can be thought of as an indirect measure of other health factors that contribute to brain atrophy in older adults. In other words, brain age accounts for a unique portion of variability not accounted for by chronological age. For instance, a brain that "looks" younger has preserved neuroplastic repair mechanisms in place relative to chronological age, whereas an older looking brain has reduced capacity to support functional recovery relative to chronological age. For this reason, we suspect that the relationship between brain age and recovery could be less impacted by severity than is the case for chronological age. Future work, including ongoing work in our lab, needs to investigate the relationship between brain age and aphasia severity in greater detail.

In an effort to minimize the confounding effects of aphasia severity, we treated the difference between estimated brain age and chronological age as a proportion of chronological age (*proportional brain age difference*; PBAD). PBAD enables fair comparison of the difference between estimated and chronological age across all participants, irrespective of age.

10. P10, line 37-39: "Our results revealed a positive correlation between chronological age and brain age, suggesting that these two measures are strongly related. Notwithstanding, we found that brain age was associated with both language recovery when variability explained by chronological age was accounted for.": there are two different measures: estimated age and PBAD, but only one concept: 'brain age'. These two measures are probably not correlated and

lead to different results. Therefore, to distinguish them, in the ms, may need two concept terms referring to these two different measures, respectively.

Authors' Response: We thank the reviewer for this valuable comment. This is entirely correct, and we acknowledge that we made the mistake of referring to both concepts as one ("brain age"). PBAD is the measure used in all statistical analyses and we have revised the conceptual use in the manuscript as appropriate.

Center for the Study of Aphasia Recovery (C-STAR)
Arnold School of Public Health
University of South Carolina

Editorial Office comments

Authors' response: Dear Scientific Editor, thank you for your comments. We have made every effort to implement the requested changes in a satisfactory manner. All changes are highlighted in yellow in the revised manuscript. Please see responses to specific questions below.

1) FIg1 and Fig5: please provide details of the statistical analysis performed, the experimental unit (n= mice, cells, wells, plate downs, etc)) and the number of data-points for each group analysed in these plots, and if the number is <10 show the single data-points.

Authors' response:
Figure 1 – Figure 1 shows descriptive data; no statistical analyses were performed to derive this plot. We added information about the number of participants with TPO data.
Figure 5 – Relevant information from the paired *t*-test on language scores obtained at stroke onset and follow up has been added

2) Please, improve the resolution of Fig4-5.

Authors' response: The resolution of Figures 4 and 5 has been improved in the revised manuscript.

3) Please supply a graphical abstract. A graphical abstract is a visual representation of the central finding or methodology of the paper. It will be displayed in the table of contents. Authors should strive to make the graphical abstract informative, interesting, visually appealing, and straightforward (no caption needed, no abbreviations except gene names). Please upload as a separate high resolution image file (tiff preferred). The technical requirements for the graphical abstracts are:
--size of the submitted image: 1200 pixels square at 300dpi
--font of the text: Arial 12-16 point (smaller fonts will not be legible online)

Authors' response: We have added a graphical abstract emphasizing the main design and findings of our study.

3) Please add an Abbreviated Summary (up to 50 words) of your paper that captures the main purpose and conclusions of the work. The summary should state the main results written in the third person ('[1st author surname] et al. report that …') and conclusion. This summary will be used in the contents list online and should not contain any abbreviations other than those on the accepted abbreviations list and accepted gene/protein names, making it accessible to the non-specialist. Please upload it as a Word document. Allowed abbreviations: AIDS; ANOVA;

AMPA; ATP; A,T,C,G; CNS; CSF; CT; DNA; ECG; EEG; EMG; GABA; HIV; MRI; NMDA; PET; PCR; RNA.

Authors' response: An Abbreviated Summary has been added to the revised manuscript.

4) Please include a paragraph about data availability in the 'Materials and Methods' section. We strongly encourage data sharing either as supplementary information or uploads to repositories which you can link in the paper.

Authors' response: We have added a subchapter titled "Data Availability" in the Methods section.

5) To comply with our ethical standards, the appropriate checklist must be included (see Reporting Guideline section within 'Instructions to authors').

Authors' response: We have included the appropriate checklist with the revised manuscript.
