# Supplementary material for: Brain age predicts long-term recovery in post-stroke aphasia
Source: Brain Commun. 2022 Oct 6;4(5):fcac252. doi: 10.1093/braincomms/fcac252 (PMC9576153; doi:10.1093/braincomms/fcac252)
Supplement: fcac252_Supplementary_Data [file Original Submission - Decision Letter.pdf › Original Submission - Decision Letter.pdf]

Original Submission - Decision Letter

18-May-2022

Dear Mr. Kristinsson,

Thank you for your recent submission BRAINCOM-2022-082 entitled "Brain Age Predicts Long-Term Recovery in Post-Stroke Aphasia" to Brain Communications.

Your manuscript has been reviewed, and while the reviewers and editorial team appreciate many aspects of the paper, they raise some major concerns that you will need to address in order for the paper to be considered for publication.

If you can address these concerns, I invite you to submit a revised manuscript and a response to each of the reviewers' comments. The reviewer(s)' comments are included at the bottom of this message.

In addition to the reviewers' comments, please also address our Scientific Editor's comments added below to comply with our requirements for presentation of scientific data at Brain Communications.

Please format your revised manuscript according to our Instructions to Authors https://academic.oup.com/braincomms/pages/General_Instructions – a summary of key points is attached to help with this.

To revise your manuscript, click the link below or log into https://mc.manuscriptcentral.com/braincom and enter your Author Centre, where you will find your manuscript title listed under "Manuscripts with Decisions." Under "Actions," click on "Create a Revision." Your manuscript number will be appended to denote a revision.

*** PLEASE NOTE: This is a two-step process. After clicking on the link, you will be directed to a webpage to confirm. ***

https://mc.manuscriptcentral.com/braincom?URL_MASK=00514e22fcdb4a8ab12a0154a203930a

Please upload a revised manuscript with changes tracked with track changes or in coloured text.

When submitting your revised manuscript, you will be asked to respond to the reviewer(s) comments. Please be as specific as possible in your response to all of the comments.

The editorial team at Brain Communications aims to increase transparency in scientific publishing and to demonstrate the value that peer review brings to the scientific process. Please therefore consider whether you are willing to have reviewer(s)' comments on your paper and your response published as supplementary data with the paper if it is accepted. This is entirely optional and will only be done if both the authors and reviewers agree. While submitting your revision there will be an opportunity to review or update the answer you gave previously, if necessary.

IMPORTANT: Your original files are available to you when you upload your revised manuscript. Please delete any redundant files before completing the submission.

Because we are trying to facilitate timely publication of manuscripts submitted to Brain Communications, we would appreciate your revised manuscript as soon as possible. If it is not possible for you to resubmit within 3 months, the option to submit a revision will expire, and we would then consider your revised paper as a new submission. However, if you need a little more time, please get in touch and we can extend this deadline for you.

Once again, thank you for submitting your manuscript to Brain Communications and I look forward to receiving your revision.

Best wishes

Professor Alfred K. Njamnshi
Associate Editor, Brain Communications
alfred.njamnshi@brainafrica.org

Professor Tara Spires-Jones
Founding Editor, Brain Communications
Tara.Spires-Jones@ed.ac.uk

Reviewer(s)' Comments to Author:

Reviewer: 1

Comments to the Author
Kristinsson and colleagues present a new method to evaluate a usual suspect in the recovery of post-stroke aphasia, which remains elusive: brain age. The evaluation of this method in a small sample shows its feasibility and opens the door for it to be evaluated further. The studies has its limitations, which the authors acknowledge, but these should not preclude the publication of the study.

Reviewer: 2 (Junhua Ding)

Comments to the Author
In the manuscript, Kristinsson et al. aimed to use brain age predicting stroke patients' language function and long-term recovery. They collected 49 participants with left stroke. Brain age was estimated through machine learning algorithm. The multiple regression results revealed that brain age is an effective biomarker for stroke's language function and recovery, irrespective of the influence of chronological age and lesion volume.

The study has a relatively large sample size for follow-up analysis. The method is solid, the result is clear, the manuscript is well-written. Most importantly, the finding may make progress for future clinical application, as stroke prognosis is still hard to predict. Following are several concerns about it.

1. The main concern is the recovery analysis. Why did the authors look at the main effects of the predictors? If want to control for baseline severity, may need to look at the interaction between predictors and behavioral performance.

2. Whether the model including brain age is significantly improved compared with model with lesion volume only?

3. According to figure 4, many points are on the ceiling or floor for individual language tasks, making the scatter patterns odd. Do they heavily influence the result?

4. What the information can tell by splitting participants in figure 4? The authors may want to indicate younger group had better performance? But it doesn't look like that.

5. In discussion, the authors can discuss different results of individual language measures and give some explanations.

6. P7: Why did brain/chronological age negatively correlate with lesion volume? Referring to Q9, shouldn't they be positive or no significant relation? Is this a general result pattern or specific for this cohort only?

7. P5: All the participants were first-time stroke? Do they have any chronic lesions?

8. P5: may need to report the TPO of the acute cohort. In addition, why is acute lesion demarcated on FLAIR not DWI?

9. P3, line 15-16; P10, line 29-30: “older individuals are more likely to present with severe aphasia ,which may negate the direct association between age and recovery.” Is it the same situation for brain age, given brain and chronological ages are highly related? How did the study overcome this issue? Smaller age variability or using the proportional difference?

10. P10, line 37-39: “Our results revealed a positive correlation between chronological age and brain age, suggesting that these two measures are strongly related. Notwithstanding, we found that brain age was associated with both language recovery when variability explained by chronological age was accounted for.”: there are two different measures: estimated age and PBAD, but only one concept: ‘brain age’. These two measures are probably not correlated and lead to different results. Therefore, to distinguish them, in the ms, may need two concept terms referring to these two different measures, respectively.

Comments from the Scientific Editor:

- FIg1 and Fig5: please provide details of the statistical analysis performed, the experimental unit (n= mice, cells, wells, plate downs, etc)) and the number of data-points for each group analysed in these plots, and if the number is <10 show the single data-points.

- Please, improve the resolution of Fig4-5.

-Please supply a graphical abstract. A graphical abstract is a visual representation of the central finding or methodology of the paper. It will be displayed in the table of contents. Authors should strive to make the graphical abstract informative, interesting, visually appealing, and straightforward (no caption needed, no abbreviations except gene names). Please upload as a separate high resolution image file (tiff preferred). The technical requirements for the graphical abstracts are:
  --size of the submitted image: 1200 pixels square at 300dpi
  --font of the text: Arial 12-16 point (smaller fonts will not be legible online)

-Please add an Abbreviated Summary (up to 50 words) of your paper that captures the main purpose and conclusions of the work. The summary should state the main results written in the third person (‘[1st author surname] et al. report that …’) and conclusion. This summary will be used in the contents list online and should not contain any abbreviations other than those on the accepted abbreviations list and accepted gene/protein names, making it accessible to the non-specialist. Please upload it as a Word document. Allowed abbreviations: AIDS; ANOVA; AMPA; ATP; A,T,C,G; CNS; CSF; CT; DNA; ECG; EEG; EMG; GABA; HIV; MRI; NMDA; PET; PCR; RNA.

-Please include a paragraph about data availability in the 'Materials and Methods' section. We strongly encourage data sharing either as supplementary information or uploads to repositories which you can link in the paper.

- To comply with our ethical standards, the appropriate checklist must be included (see Reporting Guideline section within 'Instructions to authors').
